# Structural basis of efficacy-driven ligand selectivity at GPCRs

**DOI:** 10.1038/s41589-022-01247-5

**Published:** 2023-02-13

**Authors:** Alexander S. Powers, Vi Pham, Wessel A. C. Burger, Geoff Thompson, Yianni Laloudakis, Nicholas W. Barnes, Patrick M. Sexton, Steven M. Paul, Arthur Christopoulos, David M. Thal, Christian C. Felder, Celine Valant, Ron O. Dror

**Affiliations:** 1 Department of Chemistry, Stanford University, Stanford, CA USA; 2 Department of Computer Science, Stanford University, Stanford, CA USA; 3 Department of Molecular and Cellular Physiology, Stanford University School of Medicine, Stanford, CA USA; 4 Department of Structural Biology, Stanford University School of Medicine, Stanford, CA USA; 5 Institute for Computational and Mathematical Engineering, Stanford University, Stanford, CA USA; 6 Drug Discovery Biology, Monash Institute of Pharmaceutical Sciences, Monash University, Parkville, Victoria Australia; 7 ARC Centre for Cryo-Electron Microscopy of Membrane Proteins, Monash Institute of Pharmaceutical Sciences, Monash University, Parkville, Victoria Australia; 8 Karuna Therapeutics, Boston, MA USA; 9 Neuromedicines Discovery Center, Monash Institute of Pharmaceutical Sciences, Monash University, Parkville, Victoria Australia

**Keywords:** G protein-coupled receptors, Cell signalling, Pharmacology, Computational chemistry

## Abstract

A drug’s selectivity for target receptors is essential to its therapeutic utility, but achieving selectivity between similar receptors is challenging. The serendipitous discovery of ligands that stimulate target receptors more strongly than closely related receptors, despite binding with similar affinities, suggests a solution. The molecular mechanism of such ‘efficacy-driven selectivity’ has remained unclear, however, hindering design of such ligands. Here, using atomic-level simulations, we reveal the structural basis for the efficacy-driven selectivity of a long-studied clinical drug candidate, xanomeline, between closely related muscarinic acetylcholine receptors (mAChRs). Xanomeline’s binding mode is similar across mAChRs in their inactive states but differs between mAChRs in their active states, with divergent effects on active-state stability. We validate this mechanism experimentally and use it to design ligands with altered efficacy-driven selectivity. Our results suggest strategies for the rational design of ligands that achieve efficacy-driven selectivity for many pharmaceutically important G-protein-coupled receptors.

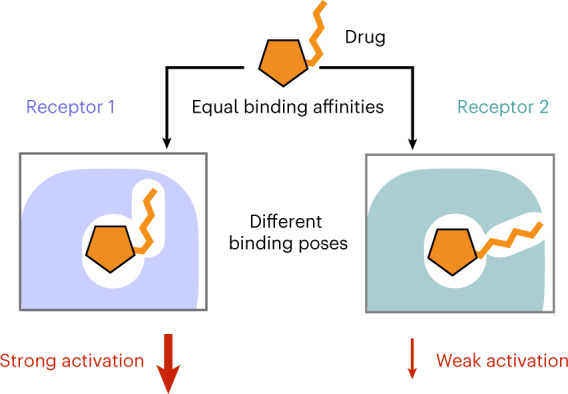

## Abstract (web summary)

The use of atomic-level simulations reveals a molecular mechanism by which a ligand can achieve selectivity between nearly identical receptors, enabling the rational design of targeted drugs.

## Main

A major challenge in drug discovery is finding selective ligands that stimulate a target receptor without stimulating off-target receptors associated with toxicity or undesirable side effects^1,2^. Most drugs achieve selectivity by binding with higher affinity to target receptors than to off-target receptors. This difference in affinities determines a range of concentrations, known as the therapeutic window, within which the drug provides desirable effects with limited undesirable effects^3^. The structural mechanisms of such binding selectivity, which we term ‘affinity-driven selectivity’, have been studied extensively^4,5^.

Over the past several decades, researchers have serendipitously discovered a number of ligands that achieve selectivity in a very different manner, which we term ‘efficacy-driven selectivity’^6–8^. Such ligands stimulate their target receptor more strongly than an off-target receptor despite binding with similar affinity to both. In other words, the ligand has higher efficacy at the target than at the off-target receptor, and it activates the target more than the off-target receptor even at saturating concentrations.

Because such a ligand does not strongly stimulate the off-target receptor even at very high concentrations, efficacy-driven selectivity can provide a much broader therapeutic window than affinity-driven selectivity under certain conditions. In addition, achieving substantial affinity-driven selectivity between closely related receptors is typically difficult when target and non-target receptors have similar binding sites, particularly when the receptors are closely related and bind the same endogenous ligands^9,10^. Efficacy-driven selectivity offers an alternative solution in such cases. Ligands with efficacy-driven selectivity have been identified for a wide range of receptor families, including muscarinic acetylcholine^6,11^, cannabinoid^8^, opioid^12,13^, dopamine^7,14^, metabotropic glutamate^15^ and nuclear hormone receptors^16^.

Unfortunately, the molecular mechanism of efficacy-driven selectivity remains unclear, severely hindering rational design of such ligands. How can a ligand differentially activate related receptors when it binds equally well to both? Efficacy-driven selectivity might result from differences in binding kinetics, receptor oligomerization, receptor internalization, biased signaling or binding to various receptor conformations. What is the key mechanism, and what is its structural basis?

To address these questions, we studied the ligand xanomeline, a muscarinic acetylcholine receptor (mAChR) agonist whose efficacy-driven selectivity has long been of pharmaceutical interest^6,11,17^. The mAChRs are targets of widely used drugs and of many current drug discovery efforts^18^. Xanomeline was initially developed over 25 years ago as a potential treatment for Alzheimer’s disease^17,19^ and recently completed a phase III clinical trial for treatment of schizophrenia^20,21^. Multiple studies over the past three decades have demonstrated that although xanomeline has nearly identical binding affinity for all five mAChR subtypes (M1–M5), it stimulates them to substantially different extents^19^. In particular, xanomeline acts as a much stronger agonist at the M4 mAChR than at the M2 and M3 mAChRs^17,22^. This property is highly desirable because stimulation of the central M4 mAChR is associated with favorable antipsychotic and cognitive effects, whereas stimulation of the peripheral M2 and M3 mAChRs is associated with cardiac and gastrointestinal side effects^11,23^.

**Fig. 1:**
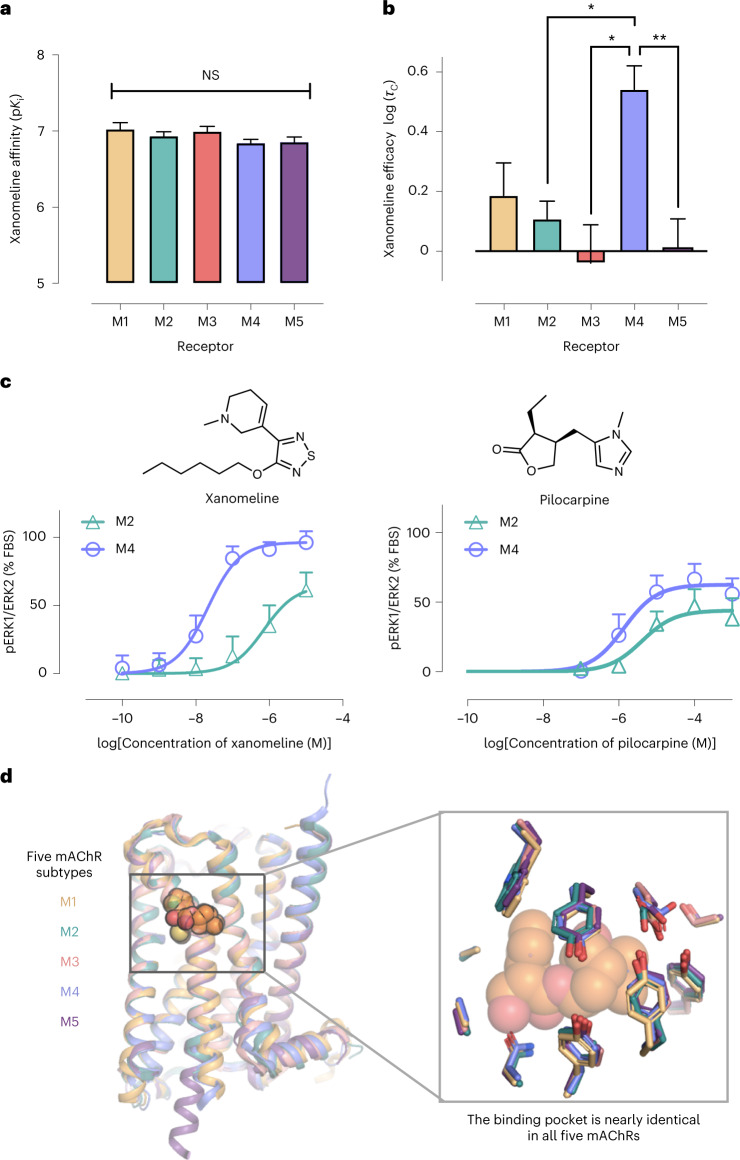
Xanomeline shows significant differences in efficacy, but not affinity, between mAChR subtypes. **a**, Xanomeline has nearly identical affinity at all mAChR subtypes, as determined by radioligand binding competition with [^3^H]*N*-methylscopolamine ([^3^H]NMS). Data are expressed as the mean ± s.e.m. from a single fit to grouped data from *n* biologically independent experiments, where *n* = 3, 4, 3, 5 and 3 for M1, M2, M3, M4 and M5 mAChRs, respectively. *P* > 0.05 for all comparisons by two-sided Tukey’s test; NS, not significant. **b**, A direct measure of ligand efficacy, log (*τ*_C_), was quantified for xanomeline across subtypes by fitting pERK1/pERK2 signaling data to an operational model of agonism and correcting for receptor expression (Methods). Xanomeline shows a significant difference in log (*τ*_C_) values between M4 and M2, M3 and M5 (*P* = 0.045, 0.024 and 0.007, respectively; *P* > 0.05 for other pairs by two-sided Tukey’s test). Data are expressed as the mean ± s.e.m. from a single fit to grouped data of *n* experiments, where *n* = 3, 4, 3, 5 and 3 for M1, M2, M3, M4 and M5, respectively. *P < 0.05; **P < 0.01. **c**, In pERK1/pERK2 signaling assays, xanomeline’s potency is greater at the M4 mAChR than at the M2 mAChR (Supplementary Table 2). The shift in xanomeline potency (left) is greater than for the control agonist pilocarpine (right), demonstrating xanomeline’s superior selectivity between M2 and M4 mAChRs. Data are plotted as the percentage of fetal bovine serum (FBS) stimulation (mean ± s.e.m.) from *n* = 6 (M2) and *n* = 9 (M4) experiments. **d**, The five human mAChR subtypes have high similarity in sequence and structure. Published crystal structures of the five mAChR subtypes (antagonist bound) are shown. Enlarged image shows that side chains within the orthosteric ligand binding pocket are identical in sequence across the receptors. The antagonist tiotropium is pictured in orange spheres for reference. Source data

Xanomeline’s selectivity is particularly noteworthy because discovery of M4-selective agonists has proven difficult^10^. Indeed, recently determined mAChR structures demonstrate that the binding pocket of the endogenous ligand acetylcholine is composed of exactly the same amino acid residues in nearly identical geometric arrangements across all five mAChR subtypes^24,25^ (Fig. 1d). Xanomeline’s selectivity for the M4 mAChR over the M2 mAChR is especially striking given that these have the highest sequence identity of any pair of mAChRs, with extremely similar overall structure and pharmacology^26^.

We thus combined computational and experimental approaches to determine the molecular mechanism of xanomeline’s efficacy-driven selectivity, initially focusing on differences between the M4 and M2 mAChRs and then considering other mAChR subtypes. Our findings shed light on the structural basis of efficacy-driven selectivity not only at mAChRs but also at other G-protein-coupled receptors (GPCRs), although the extent to which mechanisms of efficacy-driven selectivity differ across ligands and receptors remains unknown. These results may thus help guide the rational design of selective drugs for many pharmaceutically important targets.

## Results

### Xanomeline exhibits purely efficacy-driven selectivity

**Extended Data Fig. 1:**
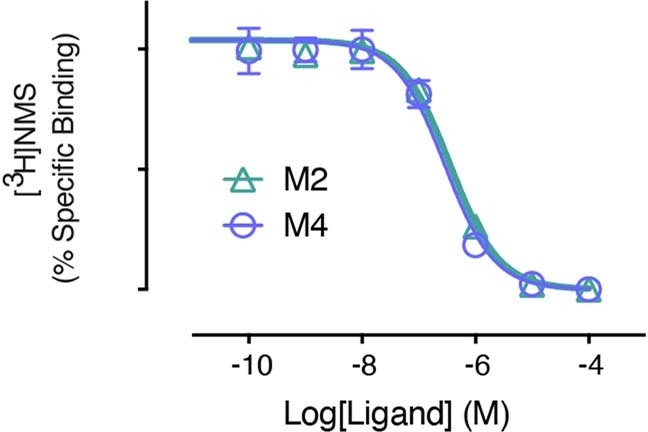
Xanomeline binds with a similar affinity to both M2 and M4 mAChRs. Binding assays were performed by titrating xanomeline and measuring competition with radiolabeled [^3^H]-N-methylscopolamine at each receptor (see Methods). Data points represent the mean ± S.E.M. from N = 4(M2), 5(M4) individual experiments. Source data

Using radioligand competition binding assays, we found that xanomeline has nearly identical binding affinity at all five mAChR subtypes (Fig. 1a, Supplementary Table 1 and Extended Data Fig. 1), in agreement with previously published studies^11,27^. To assess the signaling properties of xanomeline, we determined its ability to stimulate receptor-mediated phosphorylation of extracellular signal-regulated kinase 1 and 2 (pERK1/pERK2), a downstream signaling pathway common to all five mAChR subtypes (Fig. 1b,c and Supplementary Figs. 1 and 2). Fitting an operational model of agonism to the resulting concentration–response curves showed that xanomeline has a significantly higher efficacy at the M4 mAChR than at the M2, M3 or M5 mAChRs, even after correcting for differences in receptor expression (Fig. 1b and Methods)^28,29^. This is in contrast to the partial agonist pilocarpine, which we used as a control because xanomeline is also a partial agonist (Fig. 1c and Supplementary Tables 2 and 3). Previous studies have reported that xanomeline also has substantially higher efficacy at the M1 mAChR than at the M2, M3 and M5 mAChRs. Our measurements did lead to higher estimated efficacy at the M1 mAChR than at the M2, M3 and M5 mAChRs (Fig. 1b), but these differences were not statistically significant after correcting for receptor expression. This might also reflect the use of a signaling assay different from those of previous studies.

**Extended Data Fig. 2:**
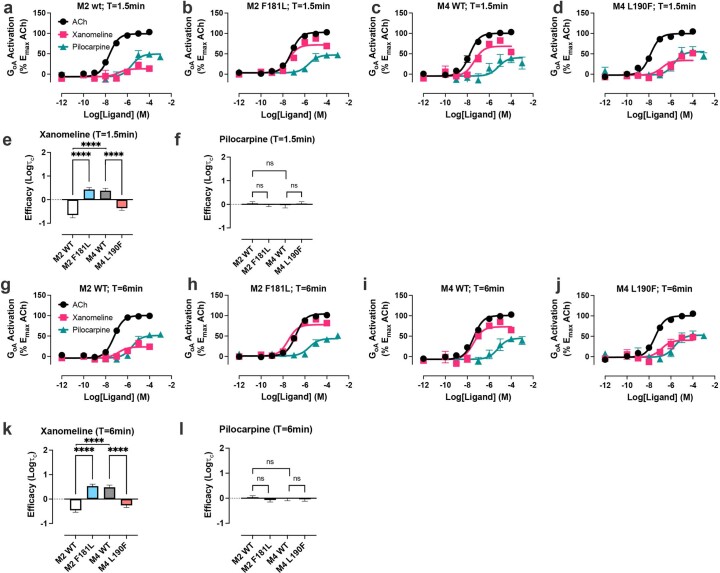
Xanomeline has higher efficacy at M4 versus M2 mAChRs in proximal assay of GoA activation. Concentration-response curve for ACh, xanomeline and pilocarpine in G_oA_ activation assay at the M2 WT **(a)**, M2 F181L **(b)**, M4 WT **(c)**, and M4 L190F **(d)** mAChRs measured 1.5 min post drug addition. Efficacy parameters quantified and corrected for receptor expression, for xanomeline **(e)** and pilocarpine **(f)**. Concentration-response curves were similar at 6 min post drug addition at M2 WT **(g)**, M2 F181L **(h)**, M4 WT **(i)**, and M4 L190F **(j)** mAChRs and for expression-corrected efficacy parameters **(k) (l)**. Data for concentration response curves are the mean ± S.E.M. of 5 experiments performed in duplicate, and normalized to the maximal response induced by ACh (% E_max_ ACh) in each cell line. Data was analyzed using the operational model of agonism to quantify the efficacy (Logτ) of each agonist in each cell line, and subsequently corrected for receptor expression level differences between cell lines (Logτ_c_), using the M2 WT as reference. Bar plots are expressed as the mean ± S.E.M. from a single fit to grouped data of N = 5 biologically independent experiments. **** indicates *P* < 0.0001 using one-way ANOVA multiple comparison analysis and Tukey’s multiple comparison test; ns (not significant) indicates *P* > .05 (Pilocarpine 1.5 min, M2 WT vs. M2 F181L: *P* = 0.72, M4 WT vs. M4 L190F: *P* = 0.72, M2 WT vs. M4 WT: *P* = 0.61, Pilocarpine 6 min, M2 WT vs. M2 F181L: *P* = 0.16, M4 WT vs. M4 L190F: *P* = 0.92, M2 WT vs. M4 WT: *P* = 0.74). Source data

To further confirm xanomeline’s efficacy-driven selectivity, we quantified its efficacy by measuring G_oA_ activation using TRUPATH biosensors at both M2 and M4 mAChRs^30^, the only mAChRs that couple predominantly to the G_i/o_ family of G proteins^11^. The results were consistent with those of the pERK1/pERK2 assay, with xanomeline displaying efficacy-driven selectivity for the M4 mAChR over the M2 mAChR (Extended Data Fig. 2).

### The active-state binding pose of xanomeline differs among mAChRs

To investigate the mechanism of efficacy-driven selectivity, we first used extensive all-atom molecular dynamics (MD) simulations to study the binding modes of xanomeline at both the active and inactive conformational states of M2 and M4 mAChRs. We placed xanomeline in the orthosteric ligand binding site (Methods), as it binds competitively with other orthosteric ligands (Extended Data Fig. 1)^31^. We performed three to ten simulations, each 2 µs in length, for each of these four conditions. We performed additional simulations with no ligand bound and with the agonist iperoxo bound (Methods).

**Fig. 2:**
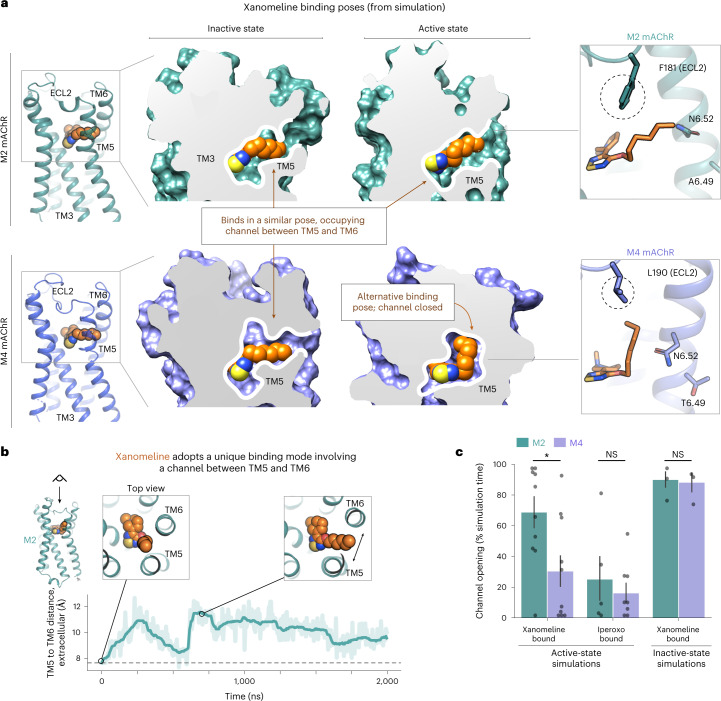
The binding mode of xanomeline differs between M2 and M4 mAChRs in the active state but not in the inactive state. **a**, Dominant binding poses of xanomeline at the inactive state (left) and active state (right) of the M2 (top) and M4 (bottom) mAChRs, as observed in MD simulations. Representative simulation snapshots are shown under each condition. Detailed images are shown on the far right. At the M4 mAChR, a smaller residue on ECL2 (M4: L190; M2: F181) allows xanomeline’s tail to extend vertically toward the extracellular vestibule, which it generally does in the active state. **b**, MD simulation trajectory showing the opening of a channel between TM5 and TM6 as measured by the distance between the extracellular ends of TM5 and TM6 (Methods). Simulations were initiated from the active-state M2 mAChR structure with xanomeline docked to the orthosteric site. The dashed line indicates the distance in the iperoxo-bound structure. Images show simulation snapshots from the indicated time points. **c**, In simulations with xanomeline bound to the active state, the TM5/TM6 channel is open much more frequently in the M2 mAChR than in the M4 mAChR (*P* = 0.027, two-sided Mann–Whitney *U*-test; *n* = 10 independent simulations; *P < 0.05). No significant difference between the M2 and M4 mAChRs was observed with xanomeline bound to the inactive state (*P* = 0.94, *n* = 3 simulations) or with control agonist iperoxo bound to the active state (*P* = 0.99, *n* = 5 and 8 simulations); NS, not significant. Data are presented as means with 68% confidence intervals (68% CIs). Source data

In these simulations, xanomeline bound very similarly to the M2 and M4 mAChRs in their inactive states. Interestingly, xanomeline’s six-carbon alkyl tail inserts between transmembrane helices 5 and 6 (TM5 and TM6; Fig. 2a,b). This binding mode requires the extracellular ends of these helices to separate slightly, forming an interhelical channel that has not been observed in experimentally determined mAChR structures with other ligands bound. This unique binding mode allows xanomeline’s tail to contact the membrane lipids, as suggested previously^32^.

**Extended Data Fig. 3:**
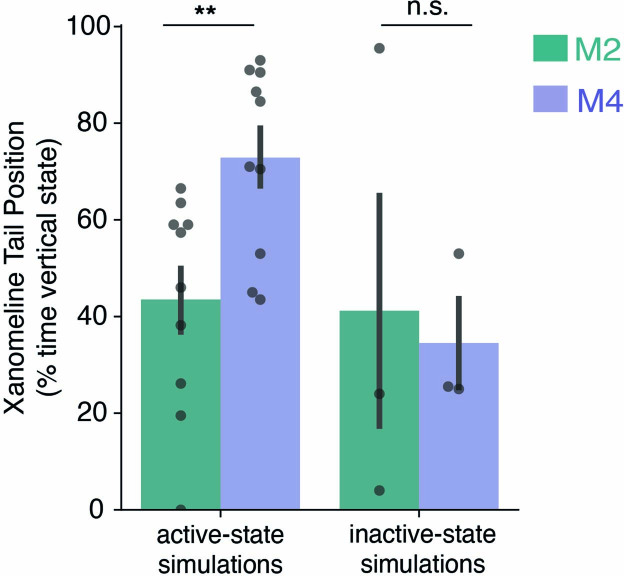
Xanomeline often adopts a different binding pose at the active state of the M2 mAChR compared to M4 mAChR. The binding pose is similar in the inactive state. Here we quantified the difference in xanomeline conformation relative to the receptor using the position of xanomeline’s alkyl tail during simulations. In each simulation frame, we categorized the tail as a vertical state or horizontal state to get the % time in a vertical state (see Methods). Bars represent the mean value from N independent simulations (M2 vs. M4 active-state *P* = 0.0032, inactive-state *P* > 0.05, two-sided Mann-Whitney U-test; N = 10 for active-state simulations and N = 3 for inactive-state simulations; error bars are 68% confidence interval). Source data

**Extended Data Fig. 4:**
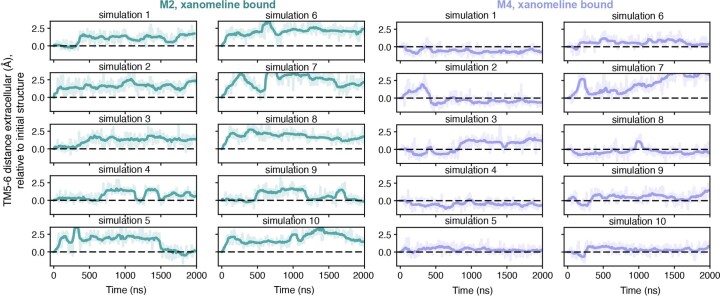
Channel opening occurs more frequently at the xanomeline-bound active state M2 than M4 mAChR. Distance between extracellular ends of TMs 5 and 6 for all xanomeline-bound active-state MD simulations of M2 and M4, illustrating that the channel opens more frequently in M2 than in M4 (see Methods). Distances are shown relative to the initial structure for each simulation (for M2, an experimentally determined structure of the active state; for M4, a template-based model of the active state). Positive values indicate an increase in distance relative to the initial structure. Source data

With the receptors in their active states, however, xanomeline’s binding mode differed substantially between the M2 and M4 mAChRs (Fig. 2a). We initiated simulations from active-state structures of the M2 and M4 mAChRs, with xanomeline docked in an identical initial pose. At the active-state M2 mAChR, xanomeline adopts a dominant pose similar to that observed in the inactive state, with its tail extending horizontally into a channel that forms between TM5 and TM6. By contrast, at the active-state M4 mAChR, xanomeline adopts a dominant pose in which its tail extends vertically toward the extracellular vestibule (Fig. 2a and Extended Data Fig. 3). This alternative pose does not require formation of a channel between TM5 and TM6, which remain closer together than at the M2 mAChR (Fig. 2c, Extended Data Fig. 4 and Supplementary Fig. 3). In simulations of the M2 and M4 mAChRs bound to iperoxo (the agonist present in the experimentally determined active-state mAChR structures), we observed no significant conformational difference between the M2 and M4 mAChR. Thus, the conformational difference observed in the presence of xanomeline is not inherent to the receptors.

**Extended Data Fig. 5:**
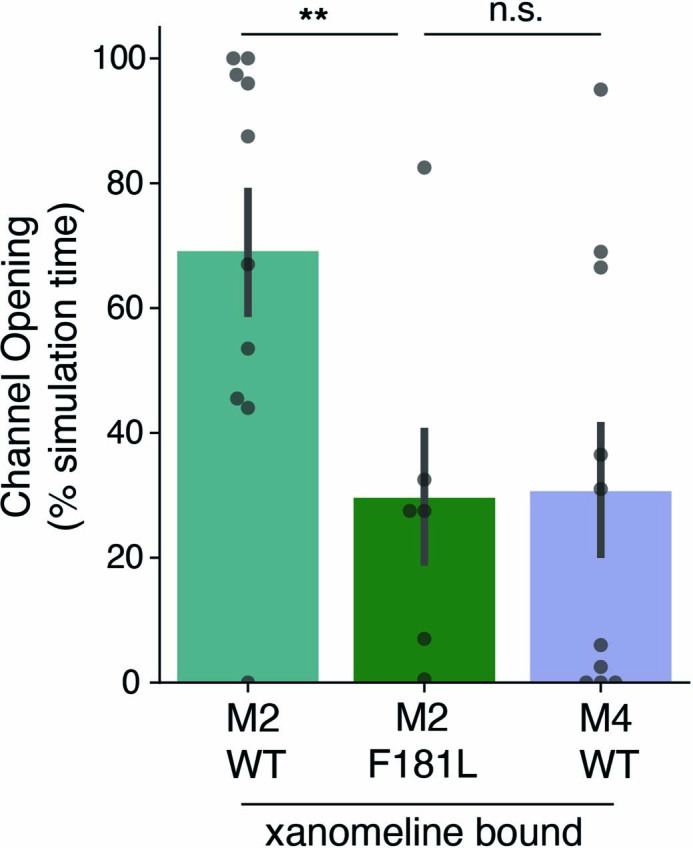
A residue on extracellular loop 2 (ECL2) is primarily responsible for the differing receptor conformation between xanomeline-bound M2 and M4 mAChRs. The M4 mAChR has a leucine (L190) at a position corresponding to phenylalanine (F181) of the M2 mAChR. In xanomeline-bound, active-state simulations, mutating residue 181 of the M2 mAChR to leucine reduces the opening frequency of the TM5/6 channel to that observed at the wild-type (WT) M4 mAChR (*P* = 0.0034 for M2 WT vs. M2 F181L; *P* > 0.05 for M2 F181L vs. M4 WT; two-sided MWU test; N = 10 independent simulations for WT and N = 6 for M2 F181L). Data presented as mean with 68% CI. Source data

Our simulations suggest that this difference in the preferred binding pose of xanomeline at the active-state M2 and M4 mAChRs is due to a sequence difference in extracellular loop 2 (ECL2), which caps xanomeline’s extended binding site. In particular, a leucine at the M4 mAChR (L190) is replaced by a phenylalanine at the corresponding position at the M2 mAChR (F181; Fig. 2a). The smaller leucine at the M4 mAChR creates an extra cavity, allowing xanomeline to easily adopt a pose with the tail extended upward, whereas the bulkier phenylalanine leaves little room for xanomeline’s tail. Xanomeline’s long tail allows the ligand to form a unique interaction with this ECL2 residue (Fig. 2a). Indeed, in simulations of the M2 mAChR with F181 mutated to leucine, xanomeline adopts the same dominant binding pose as at the M4 mAChR, with the channel between TM5 and TM6 opening as infrequently as at the M4 mAChR (Extended Data Fig. 5).

### Different binding poses lead to different efficacies

Our simulations suggest that xanomeline has similar affinity at the M2 and M4 mAChRs because it binds in very similar preferred poses to the inactive conformations of both receptors, forming essentially identical binding pocket interactions. Previous studies of mAChRs in a cellular environment have shown that the majority of the receptor population remains in the inactive state when bound to most partial agonists, including xanomeline^31,33,34^. While a small fraction of the receptor population must adopt an active conformation to bind to G proteins and stimulate signal transduction, that fraction appears to make a minimal contribution to the overall observed affinity of such ligands.

**Fig. 3:**
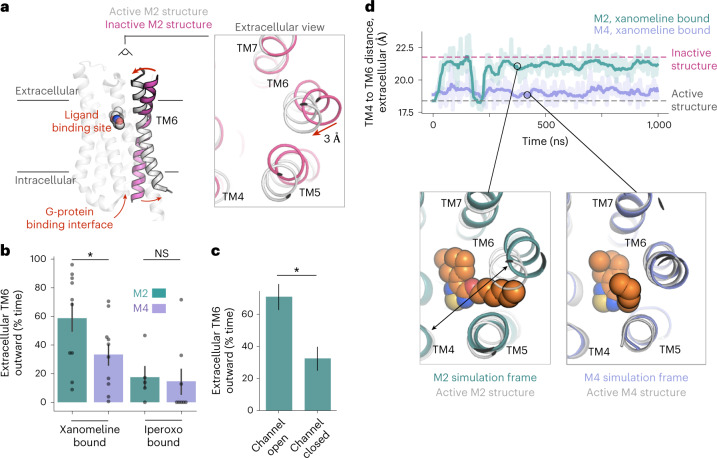
Different xanomeline binding poses lead to differing effects on TM helices and activation across receptor subtypes. **a**, At mAChRs and most other GPCRs, TM6 undergoes a large conformational change following activation, with the intracellular end of TM6 moving outward to accommodate G-protein binding and the extracellular end of TM6 moving inward toward TM4, as illustrated by experimentally determined structures of the M2 mAChR in inactive (pink) and G-protein-bound active (gray) states. **b**, Xanomeline causes the extracellular end of TM6 to be in an outward (inactive-like) conformation more often at the M2 mAChR than at the M4 mAChR (*P* = 0.037, two-sided Mann–Whitney *U*-test; *n* = 10 independent simulations; *P < 0.05), whereas no difference was observed with control agonist iperoxo bound (*P* = 0.64; *n* = 5 and 8 simulations). Data are presented as means with 68% CIs. **c**, Channel opening favors outward motion of the extracellular end of TM6, as shown for unliganded simulations of the M2 mAChR in complex with G_o_ (Methods; *P* = 0.032, two-sided Mann–Whitney *U*-test; *n* = 6 simulations; *P < 0.05). Data are presented as calculated percent from all relevant simulation frames with 68% CIs from bootstrapping; NS, not significant. **d**, In simulations initiated from active-state structures of the M2 and M4 mAChRs with xanomeline docked in an identical initial pose, the extracellular end of TM6 transitions to an inactive-like conformation at the M2 mAChR but not at the M4 mAChR. The plot shows the distance between the extracellular ends of TM6 and TM4 (corresponding to the arrow in the bottom left image) at M2 (green trace) and M4 (purple trace) mAChRs. Dashed horizontal lines show the distances in experimentally determined structures of active and inactive states of the M2 mAChR. Images show representative simulation frames (colored) overlaid on initial active-state structures (gray). Source data

Our simulations also suggest that differences in the binding pose of xanomeline at active-state M2 and M4 mAChRs lead xanomeline to favor activation more strongly at the M4 mAChR than at the M2 mAChR. Experimentally determined structures have shown that at mAChRs, as at other GPCRs, the intracellular end of TM6 moves outward on activation, making space for the G protein to bind^35^. Simultaneously, the extracellular end of TM6 moves inward toward TM3 and TM4 (Fig. 3a and Supplementary Fig. 4)^23^. In simulation, xanomeline favors this inward motion of TM6 near the ligand binding pocket more at M4 than at M2 (Fig. 3b). This difference appears to be due to the fact that opening of the channel between TM5 and TM6, which accommodates xanomeline’s tail in the horizontal pose and thus takes place more frequently at the M2 mAChR, hinders this inward motion (Fig. 3c,d). No such difference was observed in simulations of M2 and M4 mAChRs bound to the non-selective agonist iperoxo (Fig. 3b)^36^.

Our simulations thus imply that the efficacy-driven selectivity of xanomeline can be explained by a classical ternary complex model of GPCR signaling^37^ in which an agonist’s efficacy is determined by its ability to promote receptor active states over inactive states. Equivalently, an agonist has higher binding affinity to the active state than to the inactive state, and this difference in binding affinity is larger for higher-efficacy agonists. In principle, a variety of other mechanisms (involving binding kinetics, receptor internalization, receptor oligomerization or biased signaling, for example) might lead to efficacy-driven selectivity, but our simulation results imply that none of these other mechanisms are necessary to account for the selectivity of xanomeline.

Together, our results suggest not only that xanomeline binds with similar affinity to the inactive-state M2 and M4 mAChRs but also that the affinity of xanomeline increases more upon receptor activation at the M4 mAChR than at the M2 mAChR; that is, the affinity of xanomeline is greater at the active-state M4 mAChR than at the active-state M2 mAChR. G-protein-bound receptors are locked in an active state, while G-protein-free receptors primarily adopt an inactive state. We thus hypothesize that while the affinity of xanomeline for G-protein-free M2 and M4 mAChRs will be similar, the affinity of xanomeline will be greater for the G-protein-bound M4 mAChR than for the G-protein-bound M2 mAChR.

### Experimental validation of the molecular mechanism

**Fig. 4:**
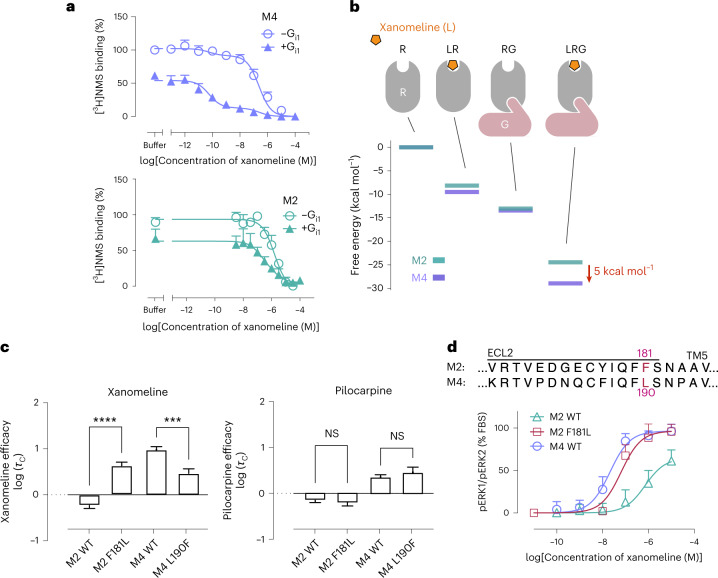
Binding and mutagenesis experiments validate computational predictions. **a**, Addition of purified G_i1_, which favors active-state receptor conformations, increases the binding affinity of xanomeline substantially more at the M4 mAChR (top) than at the M2 mAChR (bottom), as shown by competition with the radiolabeled antagonist [^3^H]NMS for purified, monomeric receptors reconstituted in lipid nanodiscs (+G_i1_ corresponds to 100 nM G_i1_). Data represent the mean ± s.e.m. from *n* = 3 independent experiments. Data were normalized to the buffer-only condition with no G protein. **b**, We determined free energies of relevant states in our model by fitting the data obtained in **a** to a ternary complex model. Cartoons at the top indicate the state (R, receptor; G, G protein), with the free energy plotted below for M2 and M4 mAChRs. As predicted, xanomeline has similar binding energies at the isolated M2 and M4 mAChRs but very different binding energies at the M2–G_i1_ and M4–G_i1_ complexes (difference of 5 kcal mol^–1^). **c**, The simulation model predicts that xanomeline efficacy differs between M2 and M4 mAChRs due to a sequence difference (leucine versus phenylalanine) on ECL2. The bar plots show efficacy corrected for receptor expression for wild-type (WT) and mutant receptors. The effect of the mutations on xanomeline efficacy aligns with the predictions of our model; F181L (M2) significantly increases efficacy (****P < 0.0001, two-sided Tukey’s test), while L190F (M4) significantly decreases efficacy (***P = 0.0005, two-sided Tukey’s test). The mutations did not significantly affect the control agonist pilocarpine (*P* = 0.70 WT M4 versus L190F and *P* = 0.93 WT M2 versus F181L, two-sided Tukey’s test). Data are expressed as means ± s.e.m. from a single fit to grouped data of *n* = 6 (M2), *n* = 6 (M2 F181L), *n* = 9 (M4) and *n* = 5 (M4 L190F) experiments; NS, not significant. **d**, In pERK1/pERK2 signaling assays, the mutation F181L (M2) increases xanomeline potency relative to the WT M2 mAChR, making the potency similar to the WT M4 mAChR. Data represent means ± s.e.m. from *n* = 6 (M2), *n* = 6 (M2 F181L) and *n* = 9 (M4) experiments. Source data

We performed several experiments to validate our computationally determined mechanism of efficacy-driven selectivity. First, we tested our hypothesis that xanomeline’s binding affinity for a G-protein-bound receptor exceeds its binding affinity for an isolated receptor by a greater margin at the M4 mAChR than at the M2 mAChR, even in a purified system where effects of oligomerization, cellular internalization, downstream signaling and interactions with other proteins can be excluded. Using M2 and M4 mAChRs reconstituted into lipid nanodiscs, we measured displacement of a radioligand across a range of xanomeline and G-protein concentrations (Fig. 4a and Supplementary Figs. 5 and 6). Increasing ratios of G_i1_ protein heterotrimer relative to receptor enhanced the ability of xanomeline to displace the radioligand, indicating successful formation of the xanomeline–G_i1_–receptor complex. At sufficient G-protein concentrations, xanomeline demonstrated two-state binding curves that allowed us to estimate its binding affinity for the G_i1_–receptor complex (the ‘high-affinity’ state) and receptor alone (the ‘low-affinity’ state). As predicted, we found that binding of G_i1_ to the receptor increases xanomeline affinity more at the M4 mAChR than at the M2 mAChR (Fig. 4a,b and Supplementary Tables 4 and 5).

Our simulations also indicate that xanomeline’s interactions differ more between active-state M2 and M4 mAChRs than between inactive-state M2 and M4 mAChRs, suggesting a larger difference in xanomeline’s binding affinity for the G_i1_–receptor complexes than for the receptors alone. The affinities determined from the nanodisc experiment align with our qualitative predictions. Whereas xanomeline’s affinities for the G-protein-free M2 and M4 mAChRs are relatively similar (p*K*_i_ = 6.12 ± 0.22 versus 6.93 ± 0.09, respectively; Supplementary Table 4), the affinities for the G_i1_–receptor complexes differ by more than 1,000-fold (p*K*_i_ = 7.10 ± 0.94 versus 10.42 ± 0.16; Fig. 4b and Supplementary Table 4).

Our simulations indicate that xanomeline’s selectivity for the M4 mAChR over the M2 mAChR reflects differences between xanomeline’s binding poses at the receptors’ active states. These differences appear to be due primarily to a sequence difference at a residue in ECL2 that caps the extended binding pocket, with a leucine residue (L190) in the M4 mAChR replaced by a phenylalanine residue (F181) in the M2 mAChR. We thus predicted that mutating L190 of the M4 mAChR to a phenylalanine would decrease the efficacy of xanomeline, while mutating F181 of the M2 mAChR to a leucine would increase the efficacy. Both mutants were generated and stably expressed in Chinese hamster ovary (CHO) cells. Subsequent quantification of agonist efficacy parameters using the pERK1/pERK2 signaling assay and the TRUPATH G_oA_ activation assay confirmed that the L190F mutation significantly decreased xanomeline’s efficacy at the M4 mAChR, and the F181L mutation significantly increased xanomeline’s efficacy at the M2 mAChR (Fig. 4c,d and Extended Data Fig. 2). Neither of these mutations had a significant effect on the efficacy of the control partial agonist pilocarpine (Fig. 4c and Extended Data Fig. 2). Moreover, in further experiments with purified receptors in nanodiscs, the F181L mutation at the M2 mAChR increased xanomeline’s binding affinity for the receptor–G-protein complex, whereas the L190F mutation at the M4 mAChR decreased binding affinity (Supplementary Table 4 and Supplementary Fig. 7).

### Rational design of ligands with altered selectivity

**Fig. 5:**
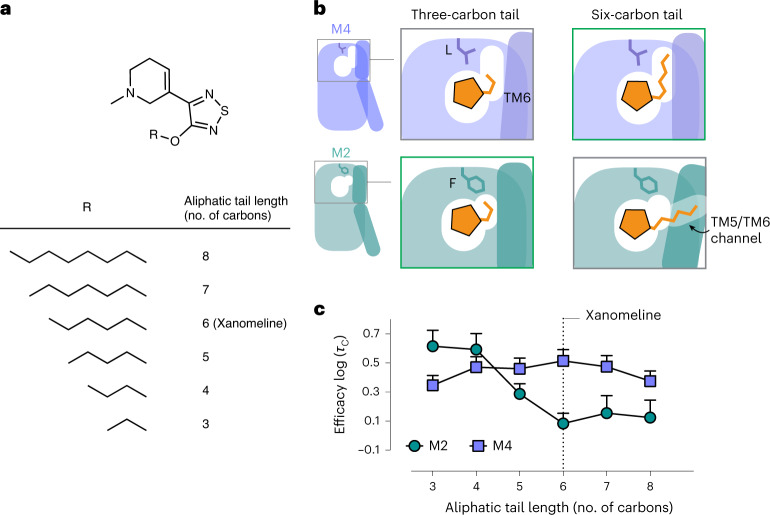
Rational design of ligands with altered efficacy-driven selectivity. **a**, To test our prediction that shortening xanomeline’s aliphatic tail would lead to an increase in ligand efficacy at the M2 mAChR relative to the M4 mAChR, we synthesized a series of xanomeline analogs with tail lengths ranging from three to eight carbons. **b**, Our model for efficacy-driven selectivity suggests that a three-carbon tail fits better into the smaller active-state M2 mAChR binding site, limiting channel opening and increasing M2 efficacy. As shown in the cartoon diagram, the three-carbon tail would be a poor fit for the more extended active-state pocket at the M4 mAChR, leaving an unfavorable gap between the tail and L181. **c**, pERK1/pERK2 signaling assays were used to measure efficacy log (*τ*_C_) of xanomeline analogs with tail lengths ranging from three to eight. As predicted, reduction in xanomeline’s tail length leads to an increase in xanomeline efficacy at the M2 mAChR relative to the M4 mAChR. The three-carbon tail molecule has efficacy-driven selectivity for the M2 mAChR over the M4 mAChR (*P* = 0.006; two-sided unpaired *t-*test), while maintaining a similar affinity for both receptors (Supplementary Table 1). Data are expressed as means ± s.e.m. from a single fit to grouped data of *n* = 4 biologically independent experiments. Source data

We sought to use our mechanistic model of xanomeline’s efficacy-driven selectivity to design ligands with altered selectivity profiles, including a ligand with efficacy-driven selectivity for the M2 mAChR over the M4 mAChR. We predicted that shortening xanomeline’s tail would increase efficacy at the M2 mAChR by allowing the ligand to bind in a tail-vertical pose without a concomitant increase in efficacy at the M4 mAChR (Fig. 5a). Shortening the tail just enough should allow it to form favorable hydrophobic packing interactions with F181 in the M2 mAChR while leaving an energetically unfavorable gap between the tail and L190 in the M4 mAChR, leading to efficacy-driven selectivity for the M2 mAChR over the M4 mAChR (Fig. 5b). These compounds have been described previously in studies of binding kinetics^38,39^, but their selectivity was not considered or tested.

Indeed, signaling assays demonstrate that shortening xanomeline’s tail dramatically increases its efficacy at M2, with no corresponding increase in efficacy at the M4 mAChR (Fig. 5c). When the tail was shortened from six carbons to three, the ligand’s efficacy at the M2 mAChR significantly exceeded its efficacy at the M4 mAChR (Fig. 5c). Our computationally derived mechanism thus enabled rational design of chemical modifications that reduced, eliminated and even reversed xanomeline’s efficacy-driven selectivity.

### A similar selectivity mechanism at other mAChRs

**Fig. 6:**
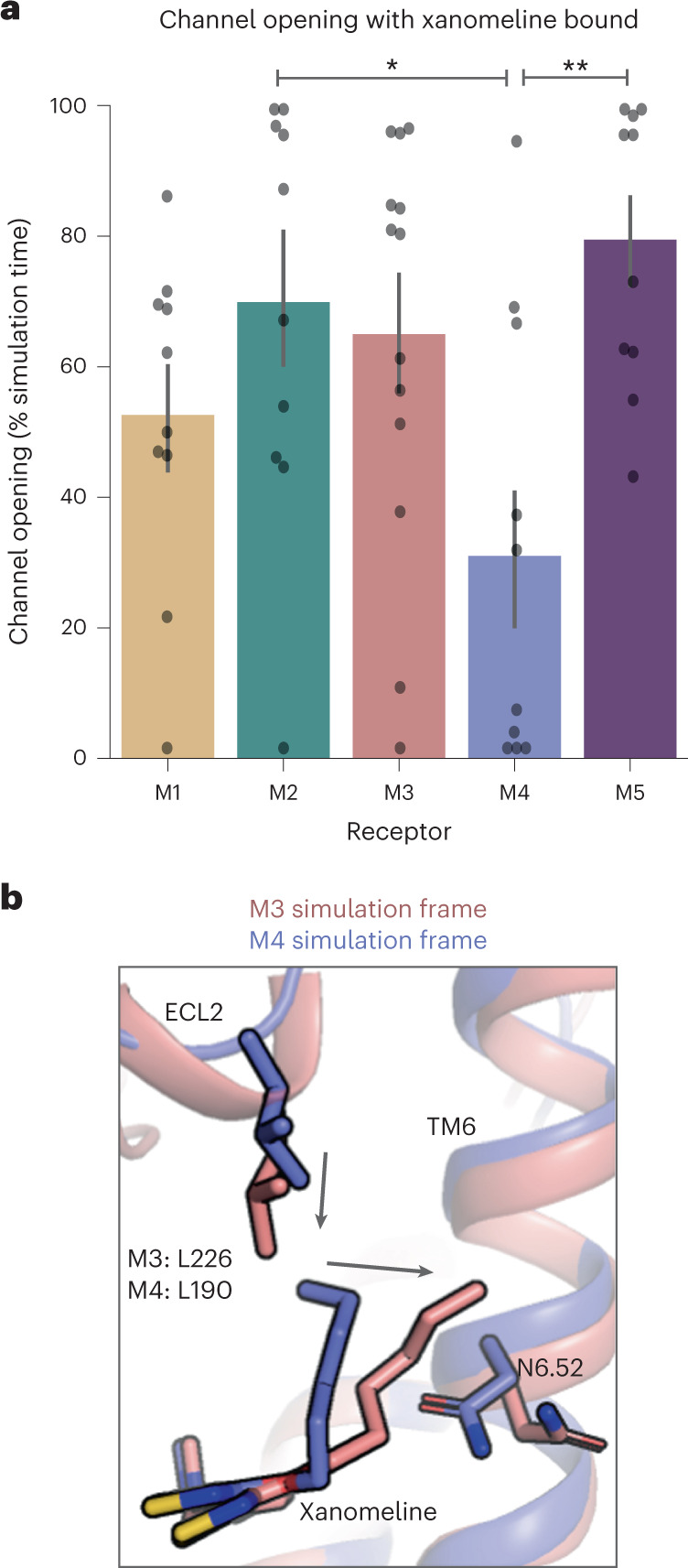
A similar mechanism explains xanomeline selectivity at other mAChR subtypes. **a**, Simulations were run for all five mAChR receptor subtypes in the active state with xanomeline bound. As at the M2 mAChR, the channel between TM5 and TM6 opens more frequently at the M3 and M5 mAChRs than at the M4 mAChR (M1 versus M4: *P* = 0.51; M2 versus M4: *P* = 0.048; M3 versus M4: *P* = 0.080; M5 versus M4: *P* = 0.008). Data were analyzed by two-sided Tukey’s test; *P < 0.05; **P < 0.01. This pattern aligns with the experimental measurements of xanomeline’s efficacy across subtypes (Fig. 1b); *n* = 13 (M3) and *n* = 10 (others) independent simulations for each condition; data are presented as means with 68% CIs. **b**, The M3 and M5 mAChRs both have a leucine corresponding to L190 (M4), but the leucine is frequently positioned further downward toward the primary binding pocket in simulations of xanomeline-bound M3 and M5 mAChRs, as illustrated here by an overlay of M3 and M4 simulation snapshots. Source data

Can this mechanism explain the pattern of xanomeline efficacy across other mAChR subtypes? We performed simulations of all five mAChRs in their active states, with xanomeline initially in the same docked pose (ten independent 2-µs simulations for each mAChR subtype). We observed a strong inverse correlation between the efficacy of xanomeline in signaling assays (Fig. 1) and the opening frequency in simulation of the TM5–TM6 channel associated with xanomeline’s horizontal binding pose (Fig. 6a and Supplementary Fig. 8). In signaling assays, xanomeline had significantly greater efficacy at the M4 mAChR than at the M3 or M5 mAChRs in addition to the M2 mAChR (Fig. 1). In simulations of the M3 and M5 mAChRs, the channel opened with a frequency more like that observed at the M2 mAChR than at the M4 mAChR (Fig. 6a). In simulation, the M1 mAChR exhibited behavior intermediate between the M4 mAChR and the M2, M3 and M5 mAChRs, in agreement with the signaling assays we used to quantify xanomeline’s efficacy.

Interestingly, the ECL2 position that we identified as a driver of M2/M4 mAChR selectivity does not explain xanomeline’s behavior at M1, M3 or M5 mAChRs, which have a leucine at this position like M4 (Supplementary Fig. 9). In fact, all the residues forming xanomeline’s extended binding pocket are identical in M1, M3, M4 and M5 mAChRs. Previous studies have suggested that the ECLs of different mAChRs differ in overall flexibility and dynamics, which may result from multiple sequence differences in the extracellular region^40,41^. In simulations of the M3 and M5 mAChRs, we observed several salt bridges in this ECL2 region that are not formed in the M4 mAChR. In the M3 mAChR, for example, E220 (ECL2) frequently forms a salt bridge with K7.32, while E5.36 forms a salt bridge with K213 (ECL2) directly above the leucine that contacts xanomeline (Supplementary Fig. 10). Formation of these salt bridges alters the conformation of ECL2 at the M3 and M5 mAChRs, favoring downward motion of the leucine and thus making it behave more like the bulky phenylalanine at the M2 mAChR (Fig. 6b and Supplementary Fig. 10). Downward motion of the leucine is also somewhat more frequent at the M1 mAChR than at the M4 mAChR, although less extreme than at the M3 or M5 mAChRs, consistent with xanomeline’s intermediate efficacy at the M1 mAChRs.

## Discussion

Efficacy-driven selectivity between closely related receptors has been observed for many drug targets, especially GPCRs, but its structural mechanism has remained unclear. Our simulations and experiments reveal the structural mechanism for the efficacy-driven selectivity of xanomeline between the five human mAChRs. We used these findings to design variants of xanomeline with altered efficacy-driven selectivity profiles, including one that reverses xanomeline’s selectivity for M2 versus M4 mAChRs.

We find that xanomeline binds in very similar modes and with similar affinities to the inactive-state conformations of all five mAChR subtypes. Xanomeline binds differently to active-state conformations of different mAChR subtypes, however, leading it to favor activation of some subtypes more than others. The changes between active-state and inactive-state binding modes are due to the contraction of mAChR binding pockets following activation. Xanomeline’s efficacy-driven selectivity for the M4 mAChR thus depends on the length of xanomeline’s tail, whose interaction with the receptor changes as the binding pocket contracts.

The mechanism we determined for efficacy-driven selectivity between mAChRs may generalize to other GPCRs. Binding pockets of many other GPCRs also contract following activation. At several GPCRs, adding a few atoms to a ligand has been found to confer or reverse efficacy-driven selectivity, suggesting that this change in binding pocket size may contribute to the selectivity. For example, diprenorphine has higher efficacy at the κ-opioid receptor than at the μ-opioid receptor, while buprenorphine, which differs only in the addition of three carbon atoms, has higher efficacy at the κ-opioid receptor than at the μ-opioid receptor, a property of substantial clinical interest^42,43^. At cannabinoid receptors, the addition of three carbon atoms to MRI2594 confers efficacy-driven selectivity for the CB1 receptor over the CB2 receptor in the resulting ligand MRI2687 (ref. ^8^). Other changes in binding pocket shape following activation could also lead to efficacy-driven selectivity via a similar mechanism.

Our findings have several implications for rational drug design, both for altering the efficacy-driven selectivity of known ligands and for discovering completely new ligands with desired efficacy-driven selectivity properties. First, we found that efficacy-driven selectivity can be explained through classical thermodynamics of binding at the orthosteric site, independent of effects such as binding kinetics, receptor internalization or receptor oligomerization. One could thus design ligands with desired efficacy-driven selectivity profiles by finding and exploiting differences in active-state structures of target and non-target receptors, for example, by screening for ligands that dock well to the active-state structure of one receptor but not another. Until recently, active-state GPCR structures were relatively rare, with most structures solved in the inactive state, but many more active-state structures are being solved thanks to advances in cryo-electron microscopy and the design of antibodies that stabilize receptors in specific conformational states^44,45^. Active-state structures can also be modeled computationally, as demonstrated in our study; our simulations of active-state M3, M4 and M5 mAChRs were based on computationally determined models.

We found that certain binding modes of xanomeline and its derivatives depend on a ‘cryptic pocket’ that has not been observed in any mAChR structure, specifically, a channel that opens between TM5 and TM6. Such cryptic pockets pose a potential challenge to structure-based drug design, but our study suggests at least two approaches to address this challenge. First, cryptic pockets often open transiently in MD simulations, even in the absence of ligands that occupy them, as in our iperoxo-bound simulations^46,47^. Second, one could discover cryptic pockets experimentally by the addition of various flexible ‘tails’ to known binders that might occupy such pockets if present.

Our results certainly do not preclude the possibility that other ligands could achieve efficacy-driven selectivity by substantially different means, particularly at other receptors. Further investigation of diverse receptors and ligands will be necessary to explore this possibility.

## Methods

### System setup for MD simulations

We performed simulations of all five mAChR subtypes with xanomeline bound to the orthosteric site and the receptor initially in the active state. These simulations did not contain an intracellular effector protein. For M2 and M4 mAChRs, we also ran active-state simulations with bound iperoxo, simulations with the apo receptor in complex with G_o_ and inactive-state simulations with xanomeline. Active-state structures were available only for the of M1 and M2 mAChRs (Protein Data Bank (PDB): 6OIJ and 6OIK, respectively). The active-state M3, M4 and M5 mAChRs were prepared using homology modeling in Prime (Schrodinger) using the M2 structure (PDB: 6OIK) as a template. Inactive-state simulations used PDB structure 3UON for the M2 mAChR and PDB structure 5DSG for the M4 mAChR. Cocrystallized T4 lysozyme, lipids, allosteric ligands and other stabilizing agents were removed where applicable. Prime (Schrodinger) was used to model in missing side chains and missing extracellular and intracellular loops, except for the long ICL3, which was not modeled in any simulations.

For active-state simulations containing bound xanomeline, the initial pose was determined using docking with Glide (Schrodinger) at the active M1 mAChR. For consistency, this initial pose was used across all active-state simulations. Our predicted xanomeline pose is supported by several observations. First, a cationic amine on xanomeline interacts with a conserved aspartate (D3.32) similar to other mAChR ligands whose binding pose is known. Second, xanomeline’s two-ring core is stable in this pose over the course of the simulation, with an average root mean squared deviation of less than 2 Å from the starting point. Simulations involving iperoxo used the pose from the cryo-electron microscopy structure of the M2 mAChR (PDB: 6OIK). For inactive-state simulations containing bound xanomeline, the initial pose was determined using docking with Glide (Schrodinger) at the inactive M2 mAChR. Standard Glide settings in Maestro were used for all docking, with the grid centered on orthosteric ligand present in the grid structure.

For all simulations, hydrogen atoms were added, and protein chain termini were capped with neutral acetyl and methylamide groups. Titratable residues were kept in their dominant protonation state at pH 7, except for D2.50 and D3.49, which were protonated (neutral) in active-state simulations, as studies indicate that these conserved residues are protonated in active-state GPCRs^48,49^. Histidine residues were modeled as neutral, with a hydrogen atom bound to epsilon nitrogen. The amine of xanomeline was protonated to form a salt bridge with the conserved aspartate in the binding site, in alignment with other muscarinic agonists. The Dowser program was used to hydrate pockets within and around each structure^50^. The receptor was then inserted into a preequilibrated palmitoyl-oleoyl-phosphatidylcholine (POPC) bilayer using Dabble^51^. Sodium and chloride ions were added to neutralize each system at a concentration of 150 mM. Approximate system dimensions were 80 Å × 80 Å × 100 Å for receptor-only simulations and 120 Å × 120 Å × 140 Å for receptor–G-protein complexes.

### Simulation protocols

All simulations were run on a single graphical processing unit using the Amber18 Compute Unified Device Architecture version of particle-mesh Ewald MD^52^. We used the CHARMM36m parameter set for protein molecules, lipids and ions and the CHARMM TIP3P water model for waters^53^. Parameters for ligands were generated using the CHARMM general force field with the ParamChem server^54^, Gaussian and AmberTools Paramfit^55^. Heating (to 310 K over 137.5 ps) and equilibration (28 ns with restraints on protein and ligand) steps were performed before production simulations^56^. Trajectory snapshots were saved every 200 ps. All simulations were at least 2 µs in length.

### Simulation analysis protocols

The AmberTools18 CPPTRAJ package^57^ was used to reimage trajectories, while visual molecular dynamics^58^, PyMol (Schrodinger) and Matplotlib Python packages were used for visualization and analysis.

In Fig. 2b, the reported distance is between the Cα atoms of M2 mAChR residues 191 (5.43) and 408 (6.56). The trajectory shows this value over the course of the simulation time, including both initial equilibration and production.

In Figs. 2c and 6a, Extended Data Fig. 5 and Supplementary Fig. 6, we report the percentage of time that the channel is formed between TM5 and TM6. To classify the receptor conformation, we observed that when the channel toward the membrane opens, N6.52 flips to a new rotamer and moves between the helices where it forms a new set of hydrogen bonds. This provides a very clear signal that is simple to threshold. This is as opposed to the distances between the TMs themselves, which are more variable due to complex three-dimensional motion; however, we note that we still get qualitatively similar results if we quantify separation directly using distance between the Cα atoms of M2 mAChR residues 191 (5.43) and 408 (6.56; Supplementary Fig. 3). To calculate a frequency, we measured the percentage of simulation time that N6.52 has a Chi 1 angle between −50 and 100. Each bar in the figures represents the mean of this value, with each simulation representing an independent sample. The threshold was determined from a visual inspection of simulation frames. We excluded the first 50 ns of simulation time in calculating the frequency. For ‘active’ conditions, only frames where the receptor remained in an active state were used. This was determined by the distance from TM3 to TM6 using the Cα atoms of residues 3.46 and 6.37; a distance greater than 10 Å was classified as active (the distance is ~8 Å in the inactive state). According to this metric, the receptors with bound xanomeline remained in an active state for approximately 50% of the 2-µs-long simulations.

In Fig. 3d, the reported distance is between the Cα atoms of M2 mAChR residues 155 (4.57) and 407 (6.55). The dashed lines are the measured distance from the available active and inactive experimental structures. We show both unsmoothed traces (thin lines) and traces smoothed with a moving average (thick lines). We use an averaging window of 30 ns.

In Fig. 3b,c, we threshold the distance plotted in Fig. 3d to calculate a percentage of simulation time that TM6 is in the outward state. A distance greater than 20.7 Å was classified as outward. For Fig. 3c, we used simulations of M2 mAChR in complex with G_o_ with no ligand bound. We then divided frames from each simulation into two groups, frames where the TM5/TM6 channel was open and frames where it was closed (as defined above). We then aggregated frames in each group and calculated the fraction of time that TM6 is in the outward state for that group

In Extended Data Fig. 3, we report the percentage of time that xanomeline’s alkyl tail is extended vertically. To calculate a frequency, we measured the distance between xanomeline C17 (located in the middle of the tail) and the Cα of a specific ECL2 residue (M2: 181; M4: 190). A distance of less than 8.9 Å was classified as the vertical state. Again, we excluded the first 100 ns of simulation time in calculating the frequency. For ‘active’ conditions, only frames where the receptor remained in an active state were used, as defined above.

The error bars for simulation results show the 68% CIs of the mean (appropriate for non-parametric distributions) calculated using bootstrapping with the Seaborn Python library. *P* values for simulation results were calculated using the Mann–Whitney *U*-test.

### Signaling assays

#### Materials

CHO FlpIn cells were purchased from Invitrogen. DMEM and FBS were purchased from ThermoTrace. Hygromycin B was purchased from Roche Applied Science. PBS and versene were made in-house. Primers used for the generation of mutant receptors were purchased from Bioneer Pacific. The AlphaScreen Surefire pERK1/pERK2 reagents were kindly provided by TGR Biosciences. AlphaScreen streptavidin donor beads, anti-IgG (protein A) acceptor beads and [^3^H]NMS (specific activity 80 Ci mmol^–1^) were purchased from PerkinElmer Life and Analytical Sciences. Xanomeline and analogs were obtained from Karuna Therapeutics. Pilocarpine hydrochloride was purchased from ICN Biomedicals. All other chemicals, including acetylcholine, were from Sigma-Aldrich.

#### Receptor mutagenesis, stable cell line generation and cell culture

Mutant receptors were generated using Q5 polymerase (New England Biolabs) in a two-step PCR protocol. Briefly, forward and reverse primers containing the desired mutation were used in separate polymerase chain reactions (25 µl each) with 100 ng of template DNA and Q5 polymerase (M2 F181L forward primer: GAGTGCTACATTCAGTTTTTGTCCAATGCTGCTGTCAC; M4 L190F forward primer: GTGCTTCATCCAGTTCTTCTCCAACCCAGCAGTG). The following reaction conditions were used: 5 min at 98 °C, then 10 cycles of 1 min at 98 °C, 1 min at 60 °C and 8 min at 68 °C plus 10 min at 68 °C before storage at 4 °C. Subsequently, both forward and reverse reactions were combined (50 µl total) with the addition of 1 µl of Q5 polymerase, and a second PCR was performed using 18 cycles of the previously described reaction conditions. DpnI restriction endonuclease (New England Biolabs) was then used to digest any remaining template DNA before transformation and confirmation of sequences (Australian Genome Research Facility). All receptor constructs (WT and mutants) were stably expressed in CHO FlpIn cells using the FlpIn Gateway technology system and selected using 600 μg ml^–1^ hygromyocin B. Cells were maintained in DMEM supplemented with 5% FBS and 600 μg ml^–1^ hygromyocin B at 37 °C in a humidified incubator (5% CO_2_, 95% O_2_).

#### Whole-cell radioligand binding assays

Saturation binding assays were first performed to estimate receptor expression and the affinity of radiolabeled [^3^H]NMS (PerkinElmer). Cells were seeded at 10,000 cells per well in 96-well white clear-bottom isoplates (Greiner Bio-one) and allowed to adhere overnight. Plates were washed once with PBS and incubated overnight at room temperature with 0.01–10 nM [^3^H]NMS in 1× HBBS binding buffer (138 mM NaCl, 5.3 mM KCl, 0.5 mM MgCl_2_, 0.4 mM MgSO_4_, 0.4 mM KH_2_PO_4_, 1.3 mM CaCl_2_, 5.5 mM d-glucose, 0.3 mM Na_2_HPO_4_ and 10 mM HEPES, pH 7.4). Equilibrium inhibition binding assays were performed to determine the affinity of the ligands. Cells were incubated overnight with increasing concentrations of each ligand in the presence of [^3^H]NMS (at the *K*_d_ concentration determined for each receptor in saturation binding). Nonspecific binding was determined by the coaddition of 10 μM atropine. After two washes with twice the original buffer volume using ice-cold 0.9% NaCl solution, cells were solubilized in 100 μl per well of Ultima Gold (PerkinElmer), and radioactivity was measured in a MicroBeta2 counter (PerkinElmer). To demonstrate that the incubation times used in our binding experiments were sufficient, we performed whole-cell competition binding experiments between [^3^H]NMS and xanomeline at each of the M1–M5 mAChRs with three incubation times: 4, 6 and 24 h (Supplementary Fig. 6). In no case did we observe a statistically significant change in the affinity estimate for xanomeline when varying incubation time.

#### pERK1/pERK2 assays

The AlphaScreen SureFire kit was used to quantify the level of pERK1/pERK2. Cells expressing the WT or mutant constructs were seeded at 20,000 cells per well into transparent 96-well plates and grown overnight at 37 °C and 5% CO_2_. Cells were washed once with PBS and incubated in serum-free DMEM at 37 °C for 4 h to allow FBS-stimulated pERK1/pERK2 levels to subside. Initial ERK1/ERK2 phosphorylation time–course experiments were performed to determine the time of the peak pERK1/pERK2 response for each ligand and each cell line (5 min for all ligands and cell lines tested; Supplementary Fig. 1). For concentration–response curves, cells were stimulated with increasing concentrations of each ligand on a heating platform at 37 °C for 5 min, the time at which the peak response was induced. For all experiments, 10% (vol/vol) FBS was used as the positive control, and vehicle controls were also performed. The reaction was terminated by removal of medium/ligands and lysis of cells with 50 μl of the SureFire lysis buffer (TGR Biosciences), and 5 μl of this lysate was transferred to a 384-well white ProxiPlate (Greiner Bio-one). Under reduced lighting conditions, a mixture of SureFire activation buffer, Surefire reaction buffer, acceptor and donor beads was prepared in a ratio of 50:200:1:1 (vol:vol:vol:vol) and added to the lysate for a lysate/mixture ratio of 5:5 (vol:vol). Plates were incubated in the dark for 1 h at 37 °C before the fluorescence signal was measured on the Envision plate reader (PerkinElmer) using standard AlphaScreen settings. To demonstrate that the responses measured in our pERK1/pERK2 assays are almost completely dependent on G_i/o_ activation at the M2 and M4 receptors, we inhibited G_i/o_ proteins through overnight treatment with 10 ng per well pertussis toxin and found that this eliminates >95% of the acetylcholine-mediated response measured in pERK1/pERK2 assays (Supplementary Fig. 2).

#### Bioluminescence resonance energy transfer TRUPATH G-protein dissociation assays

Cells were seeded at 20,000 cells per well into 96-well flat-bottom white culture plates and grown for 4 h. Plasmid DNA (20 ng per well of Gα, Gβ and Gγ at a 1:1:1 ratio) and linear polyethylenimine were added to the cells in a 1:6 ratio. To allow for protein expression, cells were then incubated (37 °C, 5% CO_2_) for 48 h before the measurement of agonist activity. Bioluminescence resonance energy transfer (BRET) assays were performed in HBSS (137 mM NaCl, 5.4 mM KCl, 0.25 mM Na_2_HPO_4_, 0.25 mM KH_2_PO_4_, 4.2 mM NaHCO_3_, 1.8 mM CaCl_2_, 0.8 mM MgSO_4_, 5.6 mM d-glucose and 10 mM HEPES, pH 7.4)^59^. Cells were incubated with 1.3 μM Prolume Purple for 5 min before reading to allow the luminescence to stabilize. BRET signals were collected using a PHERAstar FS instrument (BMG Labtech) with an emission window ratio of 515–530 nm over 410–480 nm. The initial 4 min of measurement was considered baseline counts before ligand or vehicle addition, with BRET readings every 2 min for a further 8 min following drug or vehicle addition. We measured concentration–response curves for the three selected agonists (acetylcholine, xanomeline and pilocarpine) at the M2 and M4 WT receptors and also at the M2 F181L and M4 L190F mutants. We quantified the data at two time points (1.5 and 6 min), with no qualitative difference in the findings.

### Binding assays with purified receptors in lipid nanodiscs

#### Protein expression and purification

Human M2, M4, M2 F181L and M4 L190F receptor genes were modified to contain an N-terminal Flag epitope tag and a C-terminal 8× histidine tag^24^. To increase stability and expression, ICL3 was removed. mAChR protein was expressed using the Bac-to-Bac Baculovirus Expression System (Invitrogen) in Sf9 cells. Cells were grown in ESF 921 serum-free medium (Expression System) and infected at a density of 4.0 × 10^6^ cells per ml, treated with 10 μM atropine and shaken at 27 °C for 48–60 h. Cells were collected by centrifugation (10,000*g*, 20 min, 4 °C), and cell pellets were stored at −80 °C. Cell pellets were thawed and resuspended in lysis buffer (10 mM Tris (pH 7.5), 1 mM EDTA, 1 mM MgCl_2_, 500 µM phenylmethylsulfonyl fluoride (PMSF), 1 mM leupeptin and trypsin (LT), 1 mM benzamidine, 1 mg ml^–1^ iodoacetamide, benzonase and 1 µM atropine) and stirred at 25 °C until homogenous. The cell lysate was centrifuged (10,000*g*, 15 min, 4 °C). Receptor was solubilized in solubilization buffer (30 mM HEPES (pH 7.5), 1% dodecyl-β-d-maltopyranoside (DDM), 0.2% cholate, 0.03% cholesterol hemisuccinate (CHS), 750 mM NaCl, 30% glycerol, protease inhibitors (500 µM PMSF, 1 mM LT and 1 mM benzamidine), 1 mg ml^–1^ iodoacetamide, benzonase and 1 µM atropine). The soluble fraction was separated by centrifugation (10,000*g*, 15 min, 4 °C), and the supernatant was incubated with Ni-NTA resin for 2 h at 4 °C. Ni-NTA resin was washed with wash buffer (30 mM HEPES (pH 7.5,) 0.1% DDM, 0.02% cholate, 0.003% CHS, 750 mM NaCl, 30% glycerol, 5 mM imidazole and 1 µM atropine), and protein was eluted with wash buffer supplemented with 250 mM imidazole. Sample was loaded onto M1 anti-Flag affinity resin, and detergent was exchanged from DDM solubilization buffer to lauryl maltose neopentyl glycol (LMNG) buffer (30 mM HEPES (pH 7.5), 0.01% LMNG, 0.001% CHS and 100 mM NaCl). Protein was eluted off M1 anti-Flag affinity resin through LMNG buffer supplemented with 10 mM EDTA and 0.2 mg ml^–1^ FLAG peptide. The eluate was concentrated and run through size-exclusion chromatography using a Superdex200 increase 10/300 column (GE Healthcare) with LMNG buffer. A Coomassie gel was run on fractions, and fractions containing sample were collected, concentrated to 50 mg ml^–1^, flash-frozen using liquid nitrogen and stored at −80 °C.

A covalently circularized membrane scaffold protein (MSP) construct was used (cMSP1D1), where the MSP construct was circularized during protein expression through the use of intein fragments on the N and C termini. *Escherichia coli* BL21 (DE3) cells were transformed with split intein cMSP1D1 constructs inserted into pET28a vectors (Novagen) in LB medium plus kanamycin. Induction of protein expression with 1 mM IPTG was done at an optical density at 600 nm (OD_600_) of 0.6, and cells were shaken for 16 to 20 h at 25 °C. Cells were collected by centrifugation (7,000*g*, 20 min, 4 °C), and cell pellets were stored at −80 °C. The cell pellet was resuspended in lysis buffer (50 mM Tris (pH 8), 250 mM NaCl and 0.5% Triton X-100), 0.5 mM EDTA and 1 mM PMSF were added and the cells were lysed by incubation with lysozyme for 30 min and further sonication. Lysate was dounced and put through Avestin, followed by a 30-min incubation with 2 µl of benzonase plus 10 µM benzamidine and 5 mM MgCl_2_. Cell debris were removed by centrifugation (30,000*g*, 30 min, 4 °C). A heat shock at 70 °C was conducted with the soluble fraction for 40 min. Aggregates were removed by centrifugation (30,000*g*, 30 min, 4 °C). The supernatant was loaded onto a gravity flow DEAE column. Flow-through and 5 column volumes (CV) of wash (20 mM Tris (pH 8), 320 mM NaCl and 10 mM 2-mercaptoethanol (BME)) were collected and further loaded onto a gravity flow Ni-NTA resin column (GE Healthcare). Also, flow-through and 5 CV of wash (20 mM Tris (pH 8), 320 mM NaCl, 10 mM imidazole and 10 mM BME) were collected and dialyzed to 20 mM Tris (pH 8), 0.5 mM EDTA and 10 mM BME. The urea concentration was set to 6 M, and the sample was applied to a 5-ml HiTrap QFF anion exchange column (GE Healthcare) and eluted using a 30-CV-long gradient from low-salt buffer (20 mM Tris (pH 8), 0.5 mM EDTA, 6 M urea and 10 mM BME) to high-salt buffer (20 mM Tris (pH 8), 300 mM NaCl, 0.5 mM EDTA, 6 M urea and 10 mM BME). Pure protein was pooled, dialyzed to 20 mM Tris (pH 8), 200 mM NaCl, 0.5 mM EDTA and 10 mM BME, concentrated, flash-frozen using liquid nitrogen and stored at −80 °C.

WT Gα_i1_ subunits were cloned into a PVL1392 baculovirus transfer vector. Gβ_1_ and Gγ_2_ subunits were cloned into a PVL1392 baculovirus transfer vector, with the β subunit modified to contain a C-terminal 8× histidine tag. G-protein subunits were expressed using the Bac-to-Bac Baculovirus Expression System (Invitrogen) in *Trichoplusia ni* (Hi5) insect cells. Cells were grown in ESF 921 serum-free medium (Expression System) and infected at a density of 4.0 × 10^6^ cells per ml with a 1:1 ratio of Gα to Gβγ viruses and shaken at 27 °C for 48–60 h. Cells were collected by centrifugation (10,000*g*, 20 min, 4 °C), and cell pellets were stored at −80 °C.

The cell pellet was lysed in lysis buffer (10 mM Tris (pH 7.4), 5 mM MgCl_2_, 5 mM TCEP and 10 µM GDP plus protease inhibitors (500 µM PMSF and 1 mM LT) and 1 mM benzonase). Cell lysate was centrifuged (10,000*g*, 15 min, 4 °C). Cell pellet was solubilized in 20 mM HEPES (pH 7.5), 100 mM NaCl, 1.0% sodium cholate, 0.05% DDM, 5 mM MgCl_2_, 1 mM TCEP, 10 μM GDP, protease inhibitors (500 µM PMSF, 1 mM LT and 1 mM benzamidine) and 20 mM imidazole and stirred for 60 min at 4 °C followed by centrifugation (10,000*g*, 15 min, 4 °C). The supernatant was incubated with Ni-NTA resin for 90 min at 4 °C. Resin was loaded onto glass columns and washed with wash buffer (20 mM HEPES (pH 7.5), 100 mM NaCl, 0.05% DDM, 1 mM MgCl_2_, 1 mM TCEP, 10 μM GDP and protease inhibitors (500 µM PMSF, 1 mM LT, 1 mM benzamidine and 20 mM imidazole)) until no more protein was eluting, as determined by Bradford assay. Samples were eluted with wash buffer plus 250 mM imidazole and dialyzed overnight at 4 °C to remove imidazole and to lower NaCl to 150 mM. The following morning, the sample was loaded onto 5-ml HiTrap QFF anion exchange columns (GE Healthcare) and washed with 15 CV of buffer A (20 mM HEPES (pH 7.4), 25 mM NaCl, 0.1% DDM, 1 mM MgCl_2_, 100 μM TCEP and 10 μM GDP). A gradient of 0–30% over 20 CV was then run with buffer A and buffer B (20 mM HEPES (pH 7.4), 1 M NaCl, 0.1% DDM, 1 mM MgCl_2_, 100 μM TCEP and 10 μM GDP). A Coomassie-stained SDS–PAGE gel was run on fractions, and fractions containing sample were collected, diluted with 20 mM HEPES (pH 7.4), 30 mM NaCl, 0.1% DDM, 1 mM MgCl_2_, 100 μM TCEP and 10 μM GDP to dilute NaCl to a final concentration of 125 mM. The sample was concentrated to 20 mg ml^–1^, and glycerol was added to 20%, flash-frozen using liquid nitrogen and stored at −80 °C.

#### Pharmacology

Purified M2 and M4 mAChRs were reconstituted into nanodiscs. Lipid mixture (POPG:POPC = 3:2), HNE buffer (20 mM HEPES (pH 8.0), 100 mM NaCl and 1 mM EDTA), membrane scaffold protein and receptor were mixed together to yield final concentrations of 21 mM sodium cholate, 7 mM lipid, 100 μM ApoAI and 5 μM receptor. This represents a 1:70 ratio of membrane scaffold protein to lipid, which was determined as the optimum ratio to provide a homogenous sample during nanodisc reconstitution. Following incubation on ice for 1 h, the mixture was incubated with 50 mg of Bio-Beads (Bio-Rad) per 100 µl of reconstitution mixture to remove all detergents and initiate the spontaneous formation of rHDL particles. The mixture was mixed overnight at 4 °C, and the following morning, the nanodiscs were purified through use of size-exclusion chromatography using a Superdex200 increase 10/300 column (GE Healthcare) with HNE buffer. Coomassie staining was performed on fractions, and fractions containing sample were pooled, flash-frozen using liquid nitrogen and stored at −80 °C.

The affinity of radioligand for M2 and M4 mAChRs in nanodiscs as well as the concentration of receptor in nanodiscs were determined through saturation binding with [^3^H]NMS. M2 and M4 mAChR nanodiscs were incubated with a range of concentrations of the orthosteric antagonist [^3^H]NMS in a final volume of 200 µl of assay buffer (20 mM HEPES (pH 7.5), 100 mM NaCl, 10 mM MgCl_2_ and 0.5% bovine serum albumin) for 6 h at room temperature. Nonspecific binding was determined through the use of 10 μM atropine. The assay was terminated by rapid filtration through Whatman GF/B filters using a 96-well harvester (PerkinElmer). Radioactivity was determined by the addition of 40 μl of Micro-Scint-O scintillation fluid and counting in a MicroBeta plate reader (PerkinElmer Life Sciences).

To determine the proportion of low- and high-affinity binding sites of acetylcholine and xanomeline at M2 and M4 mAChR nanodiscs, nanodiscs were incubated in a final volume of 200 μl of assay buffer containing a range of concentrations of the cold ligand in the presence of a *K*_D_ concentration of [^3^H]NMS determined through saturation binding for 6 h at room temperature. Four different molar ratios of G protein to receptor were included, where the concentration of receptor was determined through saturation binding. The four ratios were chosen based on their ability to show a progressive shift in the receptor population from a low-affinity state to a high-affinity state. The assay was terminated, and radioactivity was determined as described for saturation binding. In Fig. 4a, the concentration of G protein at M4 corresponds to an R:G_i_ ratio of 1:1,000 (G_i_ concentration of 100 nM) and at M2 correspond to an R:G_i_ ratio of 1:2,000 (G_i_ concentration of 100 nM).

### Data analysis

All graphs were analyzed using non-linear regression lines in GraphPad Prism 9.02 (GraphPad Software).

Total and nonspecific [^3^H]NMS binding data were globally fitted to a one-site saturation binding model to derive estimates of the radioligand equilibrium dissociation constant (p*K*_d_) and the maximal density of binding sites (*B*_max_; shown in Supplementary Table 6) for the human M1–M5 WT and mutant mAChRs using the following equation:

1 $$Y = \frac{{B_{\max}{\times }\left[ A \right]}}{{\left[ A \right] + K_{\mathrm{A}}}} + NS \times \left[ A \right],$$

where *Y* is radioligand binding, *B*_max_ is the total number of receptors, [*A*] is the radioligand concentration, *K*_A_ is the equilibrium dissociation constant of the radioligand and *NS* is the fraction of nonspecific radioligand binding. Radioligand inhibition binding data were empirically fitted to a one-site inhibition mass action curve to determine inhibitor potency (pIC_50_) estimates, which were then converted to p*K*_*i*_ values as appropriate.

For radioligand inhibition in nanodisc experiments in the presence of G protein, specific binding of each orthosteric ligand was fitted to a two-site binding equation:

2 $$\begin{array}{l}Y = \left(\mathrm {Top - Bottom} \right) \times \left( {\frac{{{\mathrm{fraction}}\_1}}{{1 + 10^{\left( {{{{\mathrm{log}}}}\left[ B \right] - {\mathrm{logIC}}_{50\_1}} \right)}}} + \frac{{1 - {\mathrm{fraction}}\_1}}{{1 + 10^{\left( {{{{\mathrm{log}}}}\left[ B \right] - {\mathrm{logIC}}_{50\_2}} \right)}}}} \right) \\+ \mathrm {Bottom},\end{array}$$

where Top and Bottom represent the maximal and minimal asymptotes of the curve, respectively, logIC_50_ is the logarithm of the concentration of inhibitor that reduces half the maximal radioligand binding for each binding site, log[*B*] is the concentration of inhibitor and fraction_1 is the proportion of high-affinity binding sites. LogIC_50_1_ corresponds to high-affinity binding sites, and logIC_50_2_ corresponds to low-affinity binding sites. IC_50_ values were converted to the low- and high-affinity equilibrium dissociation constants (p*K*_i low_ and p*K*_i high_) using the Cheng and Prusoff equation^60^.

Concentration–response curves were fitted to a three-parameter logistic equation to derive ligand potency (pEC_50_) estimates. Agonist concentration–response curves were also fitted to an operational model of agonism to estimate efficacy in the system (log*τ*)^28^ using the following equation:

3 $$Y = {\mathrm{Basal}} + \frac{{\left( {\mathrm{{Em}}\, -\, {\mathrm{Basal}}} \right)}}{{1 + \frac{{10^{{\mathrm{log}}K_{\mathrm{A}}} + 10^{{\mathrm{log}}\left[ A \right]}}}{{10^{{\mathrm{log}}\tau }\times 10^{{\mathrm{log}}\left[ A \right]}}}}},$$

where Em is the maximal possible response of the system (not the agonist), Basal is the basal level of response in the absence of agonist, *K*_A_ denotes the functional equilibrium dissociation constant of the agonist A and *τ* is an index of the efficacy of the agonist and is defined as *R*_T_/*K*_E_ (where *R*_T_ is the total concentration of receptors, and *K*_E_ is the concentration of agonist–receptor complex that yields half the maximum system response (Em)). To define the Em and *τ* for each mutant and assay, the *K*_A_ for all high-efficacy agonists was constrained to equal the *K*_i_ value derived from radioligand binding assays in the non-linear regression procedure. Both theory and experimental evidence have shown that *τ* varies linearly with receptor expression, whereas other measures, such as pEC_50_ and the activity ratio, do not^29^. To correct ligand efficacy estimates for differences in receptor expression (Supplementary Table 6) between two receptors expressed in two different cell lines (receptor A and receptor B), we take *τ* at receptor B and normalize it by multiplying by the ratio of receptor B expression to receptor A expression to yield *τ*_C_ (ref. ^29^).

All affinities, potencies and cooperativity parameters were estimated as logarithms. All results are expressed as the means ± s.e.m. from a single fit to grouped data computed in Prism. Statistical tests (one-way analysis of variance and Dunnett’s tests) were performed as appropriate.

### Reporting summary

Further information on research design is available in the Nature Portfolio Reporting Summary linked to this article.

## Online content

Any methods, additional references, Nature Portfolio reporting summaries, source data, extended data, supplementary information, acknowledgements, peer review information; details of author contributions and competing interests; and statements of data and code availability are available at 10.1038/s41589-022-01247-5.

## Supplementary information

Supplementary Information Supplementary Methods, Tables 1–6 and Figs. 1–10.

Reporting Summary

## Data Availability

Simulation trajectories are available at 10.5281/zenodo.7407352. Structural models used in this study were accessed from the PDB under accession codes 6OIJ (M1, G_11_), 6OIK (M2, G_o_), 3UON (M2) and 5DSG (M4). Source data are provided with this paper.

## Source data

Source Data Fig. 1 Statistical source data.
Source Data Fig. 2 Statistical source data.
Source Data Fig. 3 Statistical source data.
Source Data Fig. 4 Statistical source data.
Source Data Fig. 5 Statistical source data.
Source Data Fig. 6 Statistical source data.
Source Data Extended Data Fig. 1 Statistical source data.
Source Data Extended Data Fig. 2 Statistical source data.
Source Data Extended Data Fig. 3 Statistical source data.
Source Data Extended Data Fig. 4 Statistical source data.
Source Data Extended Data Fig. 5 Statistical source data.
