# Author Correction: Structural basis of efficacy-driven ligand selectivity at GPCRs

**DOI:** 10.1038/s41589-023-01297-3

**Published:** 2023-03-06

**Authors:** Alexander S. Powers, Vi Pham, Wessel A. C. Burger, Geoff Thompson, Yianni Laloudakis, Nicholas W. Barnes, Patrick M. Sexton, Steven M. Paul, Arthur Christopoulos, David M. Thal, Christian C. Felder, Celine Valant, Ron O. Dror

**Affiliations:** 1 Department of Chemistry, Stanford University, Stanford, CA USA; 2 Department of Computer Science, Stanford University, Stanford, CA USA; 3 Department of Molecular and Cellular Physiology, Stanford University School of Medicine, Stanford, CA USA; 4 Department of Structural Biology, Stanford University School of Medicine, Stanford, CA USA; 5 Institute for Computational and Mathematical Engineering, Stanford University, Stanford, CA USA; 6 Drug Discovery Biology, Monash Institute of Pharmaceutical Sciences, Monash University, Parkville, Victoria Australia; 7 ARC Centre for Cryo-Electron Microscopy of Membrane Proteins, Monash Institute of Pharmaceutical Sciences, Monash University, Parkville, Victoria Australia; 8 Karuna Therapeutics, Boston, MA USA; 9 Neuromedicines Discovery Center, Monash Institute of Pharmaceutical Sciences, Monash University, Parkville, Victoria Australia

**Keywords:** G protein-coupled receptors, Cell signalling, Pharmacology, Computational chemistry

Correction to: *Nature Chemical Biology* 10.1038/s41589-022-01247-5. Published online 13 February 2023.

In the version of this article initially published, Nicholas Barnes was not properly acknowledged as a co-author. He has now been included in the current author list, and his contributions are specified in the author contributions statement. The error has been corrected in the HTML and PDF versions of the article.

