# Supplementary material for: Structural basis of efficacy-driven ligand selectivity at GPCRs
Source: Nat Chem Biol. 2023 Feb 13;19(7):805–14. doi: 10.1038/s41589-022-01247-5 (PMC10299909; doi:10.1038/s41589-022-01247-5)
Supplement: Supplementary file 1 — Supplementary Methods, Tables 1–6 and Figs. 1–10. [file 41589_2022_1247_MOESM1_ESM.pdf]

nature chemical biology

Article https://doi.org/10.1038/s41589-022-01247-5

Structural basis of efficacy-driven ligand selectivity at GPCRs

In the format provided by the authors and unedited

Supplementary Table 1. Ligand binding affinities (pK_I) at mAChRs determined with radioligand binding competition

	Xanomeline	Pilocarpine	Acetylcholine	3-carbon tail analog of xanomeline
**M1**	7.03 ± 0.09 (3)	5.22 ± 0.06 (3)	4.54 ± 0.06 (3)	7.56 ± 0.05 (3)
**M2**	6.94 ± 0.07 (4)	5.33 ± 0.07 (4)	5.77 ± 0.07 (4)	7.64 ± 0.07 (4)
**M3**	7.00 ± 0.07 (3)	5.67 ± 0.06 (3)	4.50 ± 0.08 (3)	7.46 ± 0.07 (3)
**M4**	6.84 ± 0.05 (5)	4.77 ± 0.07 (5)	4.50 ± 0.05 (5)	7.43 ± 0.05 (5)
**M5**	6.86 ± 0.07 (3)	5.69 ± 0.07 (3)	4.78 ± 0.07 (3)	7.31 ± 0.06 (3)
**M2**	6.72 ± 0.11 (5)	5.47 ± 0.11 (5)	5.29 ± 0.12 (5)	
**M2(F181L)**	6.95 ± 0.13 (3)	5.38 ± 0.08 (3)	4.97 ± 0.14 (3)	
**M4**	6.84 ± 0.10 (3)	4.92 ± 0.06 (3)	4.46 ± 0.07 (3)	
**M4(L190F)**	6.63 ± 0.15 (3)	5.34 ± 0.09 (3)	4.57 ± 0.12 (3)	

Values are the negative logarithm of the equilibrium dissociation constant (pK_I) determined by radioligand binding competition with [^{3}H]-N-methylscopolamine (NMS). Data are expressed as the mean ± S.E.M. from a single fit to grouped data from (N) biologically independent experiments. The table is divided into two sets of experiments (top 5 rows and bottom 4 rows), which were conducted at different times and with different batches of cells.

Supplementary Table 2. Ligand functional parameters (pEC_{50} and E_{max}) from pERK1/2 signaling assay

	**Xanomeline pEC_{50}**	**Pilocarpine pEC_{50}**	**Acetylcholine pEC_{50}**
**M1**	7.49 ± 0.21 (3)	6.85 ± 0.16 (3)	8.32 ± 0.18 (3)
**M2**	6.23 ± 0.07 (4)	4.84 ± 0.09 (4)	7.48 ± 0.06 (4)
**M3**	6.93 ± 0.26 (3)	6.84 ± 0.20 (3)	7.43 ± 0.21 (3)
**M4**	7.15 ± 0.11 (5)	5.28 ± 0.22 (5)	7.39 ± 0.11 (5)
**M5**	6.26 ± 0.20 (3)	5.64 ± 0.26 (3)	8.15 ± 0.19 (3)
**M2**	6.14 ± 0.15 (6)	5.39 ± 0.17 (6)	7.52 ± 0.07 (6)
**M2(F181L)**	7.00 ± 0.11 (6)	5.17 ± 0.09 (6)	7.47 ± 0.08 (6)
**M4**	7.68 ± 0.06 (9)	5.87 ± 0.10 (9)	8.08 ± 0.05 (8)
**M4(L190F)**	6.81 ± 0.10 (5)	6.04 ± 0.12 (5)	8.50 ± 0.10 (4)
	**Xanomeline E_{max}**	**Pilocarpine E_{max}**	**Acetylcholine E_{max}**
**M1**	79 ± 5 (3)	83 ± 4 (3)	94 ± 4 (3)
**M2**	76 ± 3 (4)	80 ± 3 (4)	101 ± 3 (4)
**M3**	62 ± 6 (3)	64 ± 3 (3)	98 ± 5 (3)
**M4**	92 ± 4 (5)	71 ± 5 (5)	102 ± 4 (5)
**M5**	80 ± 8 (3)	74 ± 6 (3)	90 ± 4 (3)
**M2**	67 ± 6 (6)	46 ± 3 (6)	101 ± 3 (6)
**M2(F181L)**	100 ± 8 (6)	35 ± 5 (6)	101 ± 6 (6)
**M4**	101 ± 3 (9)	66 ± 3 (9)	99 ± 5 (8)
**M4(L190F)**	90 ± 8 (5)	77 ± 6 (5)	94 ± 8 (4)

Values in the top half of the table are negative logarithm of EC_{50} and values in the bottom half are E_{max} (% FBS stimulation) determined by fitting three-parameter logistic equation to concentration-response curve from pERK1/2 signaling assays (see Methods). Xanomeline and pilocarpine behaved as partial agonists relative to acetylcholine, with lower E_{max}. data are expressed as the mean ± S.E.M. from a single fit to grouped data from (N) biologically independent experiments. The table is divided into two sets of experiments (top 5 rows and bottom 4 rows) which were conducted at different times and with different batches of cells.

Supplementary Table 3. Efficacy corrected for receptor expression levels (Logτ_C), determined from fitting operational model to pERK1/2 data

	Xanomeline	Pilocarpine	Acetylcholine
M1	0.19 ± 0.11 (3)	1.5 ± 0.08 (3)	3.49 ± 0.15 (3)
M2	0.11 ± 0.06 (4)	0.24 ± 0.06 (4)	1.99 ± 0.07 (4)
M3	-0.04 ± 0.13 (3)	0.21 ± 0.13 (3)	3.12 ± 0.18 (3)
M4	0.55 ± 0.08 (5)	0.3 ± 0.07 (5)	2.94 ± 0.10 (5)
M5	0.01 ± 0.10 (3)	0.24 ± 0.11 (3)	3.43 ± 0.16 (3)
M2	-0.23 ± 0.07 (6)	-0.15 ± 0.04 (6)	1.73 ± 0.08 (6)
M2 (F181L)	0.58 ± 0.09 (6)	-0.29 ± 0.09 (6)	2.49 ± 0.10 (6)
M4	0.98 ± 0.08 (9)	0.41 ± 0.06 (9)	3.59 ± 0.08 (8)
M4(L190F)	0.46 ± 0.10 (5)	0.52 ± 0.10 (5)	3.82 ± 0.14 (4)

Values are Logτ_C determined by fitting an operational model of agonism to pERK1/2 signaling data (see Methods). Data are expressed as the mean ± S.E.M. from a single fit to grouped data from (N) biologically independent experiments. Xanomeline and pilocarpine behaved as partial agonists relative to acetylcholine, with lower Logτ_C. The table is divided into two sets of experiments (top 5 rows and bottom 4 rows), which were conducted at different times and with different batches of cells. Adjusted P-values from Tukey's multiple comparison test for xanomeline efficacies from top 5 rows are: M1 vs. M2: 0.98, M1 vs. M3: 0.70, M1 vs. M4: 0.22, M1 vs. M5: 0.70, M2 vs. M3: 0.88, M2 vs. M4: 0.045, M2 vs. M5: 0.91, M3 vs. M4: 0.023, M3 vs. M5: 0.99, M4 vs. M5: 0.007.

Supplementary Table 4. Affinity estimates for ACh and xanomeline at M2 and M4 mAChRs reconstituted in nanodiscs in the presence of G_{i1} protein heterotrimer

		M2	M4	M2 F181L	M4 L190F
**Acetylcholine**	**p$K_{I\ low}$**	6.18 ± 0.12 (4)	5.78 ± 0.05 (7)	n.a.	n.a.
	**p$K_{I\ high}$**	7.95 ± 0.23 (4)	8.64 ± 0.08 (7)	n.a.	n.a.
**Xanomeline**	**p$K_{I\ low}$**	6.12 ± 0.22 (3)	6.93 ± 0.09 (3)	6.11 ± 0.13 (3)	5.46 ± 0.11 (3)
	**p$K_{I\ high}$**	7.10 ± 0.94 (3)	10.42 ± 0.16 (3)	9.20 ± 0.20 (3)	7.83 ± 0.20 (3)

p$K_{I\ low}$ and p$K_{I\ high}$ are negative logarithm of the low- and high-affinity equilibrium dissociation constants (see Methods). Data represent the best fit value ± standard error from nonlinear regression using data from (N) individual experiments performed in duplicate. Receptor : G protein molar ratio was 1:1000. "n.a.", not available; these data were not collected.

Supplementary Table 5. Fraction of receptors in a G protein-dependent high-affinity state

	Receptor : G protein Molar Ratio					
	**1:0**	**1:50**	**1:125**	**1:500**	**1:1000**	**1:2000**
**Acetylcholine M2 mAChR**	0.13 ± 0.10	0.33 ± 0.10	n.a.	n.a.	0.57 ± 0.11	0.89 ± 0.12
**Acetylcholine M4 mAChR**	0.00 ± 0.04	n.a.	0.30 ± 0.04	0.71 ± 0.03	0.81 ± 0.04	n.a.
**Xanomeline M2 mAChR**	0.00 ± 0.27	n.a.	n.a.	0.00 ± 0.29	0.17± 0.33	0.59 ± 0.60
**Xanomeline M4 mAChR**	0.10 ± 0.04	n.a.	0.20 ± 0.04	0.66 ± 0.05	0.76 ± 0.06	n.a.

Fraction of receptors in the high-affinity binding state as determined by radioligand competition binding with agonist. Data represent the best fit value ± standard error from nonlinear regression using data from 4 to 7 individual experiments performed in duplicate. "n.a.", not available; these data were not collected.

Supplementary Table 6. Receptor expression in CHO cells used in this study.

	**Receptor Expression (B_{max}, sites/cell)**
**M1**	1610 ± 29
**M2**	440 ± 26
**M3**	1078 ± 31
**M4**	788 ± 30
**M5**	1035 ± 43
**M2**	415 ± 31
**M2(F181L)**	399 ± 44
**M4**	395 ± 26
**M4(L190F)**	522 ± 40

Expression levels were determined from saturation binding assays performed on each cell line, by defining the B_{max} (maximal number of binding sites per cell). Data represent the mean ± S.E.M. of 3 independent experiments performed in duplicate. The table is divided into two sets of experiments (top 5 rows and bottom 4 rows), which were conducted at different times and with different batches of cells.

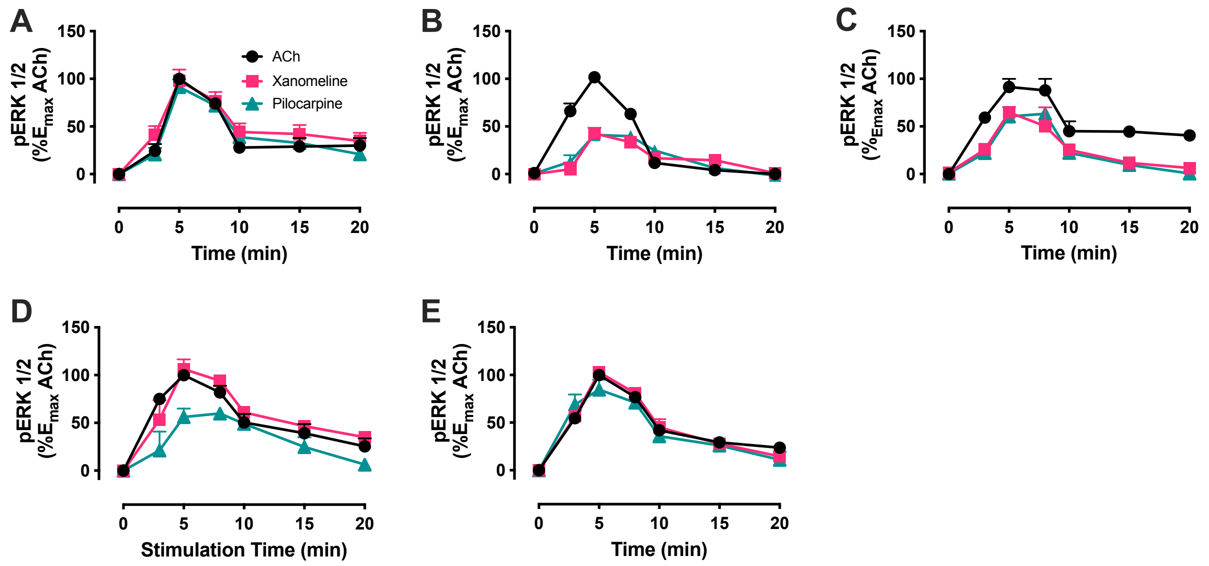

Supplementary Figure 1. Time course experiments measuring the time at which each ligand induced a maximal pERK1/2 response. CHO cells stably expressing the M1 **(A)**, M2 **(B)**, M3 **(C)**, M4 **(D)**, and M5 **(E)** mAChRs were stimulated for a period of 20 min to determine the time at which the response to each agonist (acetylcholine, xanomeline, and pilocarpine) peaked. All agonists displayed their maximal functional readout at ~5 min. Data represent the mean ± S.E.M. of N=2– experiments performed in duplicate. Data were normalized to the maximal response induced by 1 mM ACh in each cell line.

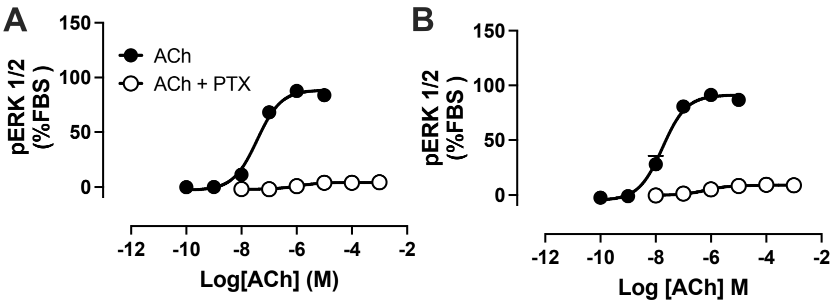

Supplementary Figure 2. ERK1/2 phosphorylation response mediated by M2 and M4 mAChRs is almost completely Gi/o-dependent. CHO cells stably expressing the M2 **(A)** or M4 **(B)** mAChRs, treated overnight with vehicle or 10 ng/well of pertussis toxin (PTX), were stimulated with increasing concentrations of ACh. Data represent the mean ± S.E.M. of 4 experiments performed in duplicate. Data were normalized to the maximal response induced by FBS (% FBS) in each cell line. Data was analyzed using a three-parameter logistic equation to quantify potency (pEC_{50}) and maximal response (E_{max}) of ACh.

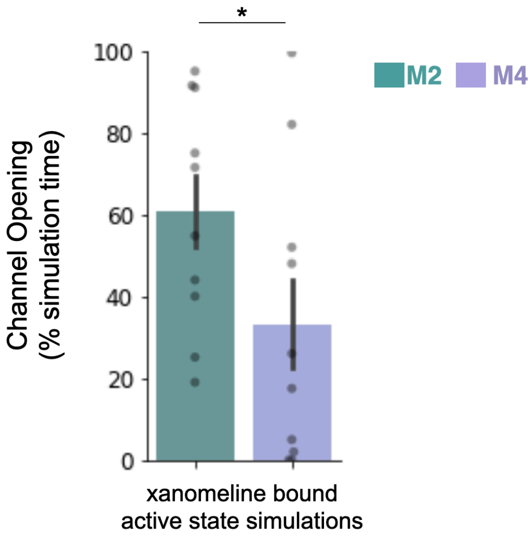

Supplementary Figure 3. Channel opening quantified using the distance metric. Channel opening is significantly different (*P*=0.044, two-sided Mann-Whitney U test, N=10 independent simulations) between M2 and M4 with xanomeline bound when quantified using distances between Cα atoms of residues 5.43 and 6.56. Data presented as mean with 68% CI.

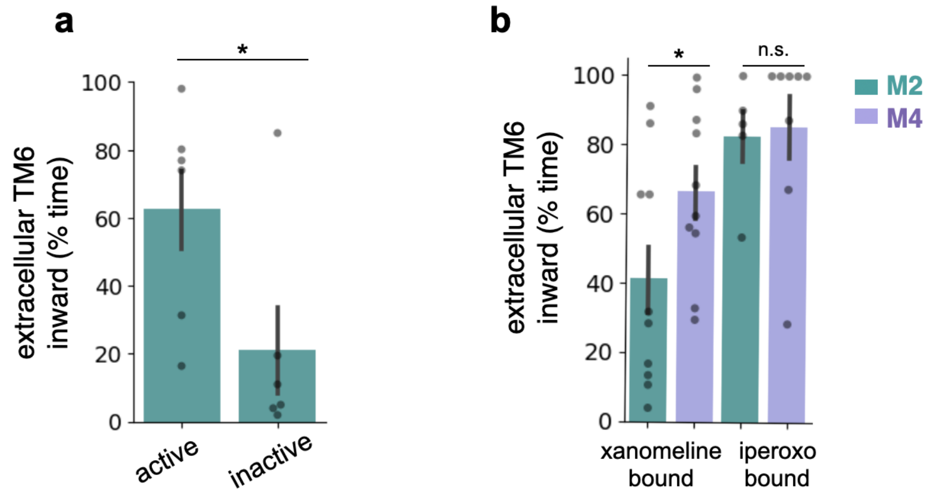

Supplementary Figure 4. Effect of receptor activation state and ligand binding on inward motion of TM6 near the ligand binding pocket (i.e., the extracellular end of TM6). (a) The extracellular end of TM6 tends to move inward upon receptor activation, as illustrated here for simulations of an unliganded M2 receptor in active and inactive states (and as previously observed in experimentally determined structures of muscarinic receptors and other GPCRs). (P=0.032, two-sided MWU test, N=6 independent simulations). Data presented as mean with 68% CI. (b) Xanomeline binding favors this inward motion of TM6 more at M4 than at M2 (P=0.037, two-sided MWU test, N=10 independent simulations). Binding of iperoxo (a stronger agonist that does not display efficacy-driven selectivity), on the other hand, favors inward motion at M2 as much as at M4 (P>0.05, N=5, 8). Data are from molecular dynamics simulations of the active-state receptor. Data presented as mean with 68% CI.

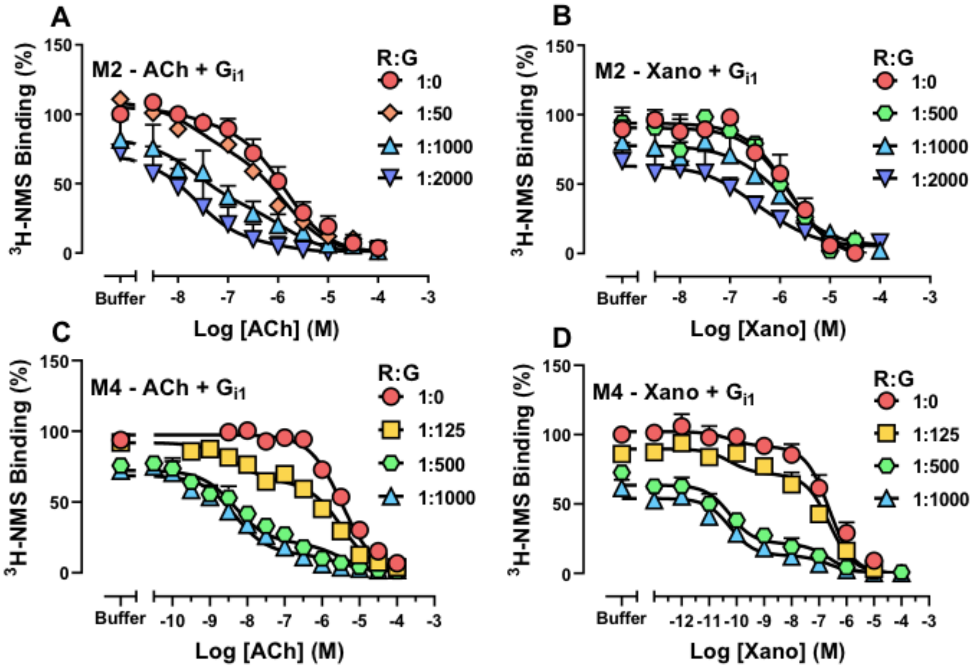

Supplementary Figure 5. Ability of acetylcholine and xanomeline to stabilize the high affinity state of the M2 and M4 mAChRs in the presence of increasing amounts of WT G_{i1} protein heterotrimer. A) Increasing ratios of WT G_{i1} protein heterotrimer relative to M2 mAChR reconstituted into nanodiscs enhances the ability of acetylcholine to compete for binding with the radioligand. This is indicative of a shift in the relative proportion of receptors in the low affinity state and the high affinity state in the presence of G protein and orthosteric agonist, where ACh has higher affinity for the high affinity state. The ability of the WT G_{i1} protein heterotrimer to create the high affinity state is detrimental to antagonist radioligand binding and as such there is a reduction in ^{3}H-NMS binding with increasing concentrations of the WT G_{i1} protein heterotrimer. **B)** At the M2 mAChR, xanomeline is a weak partial agonist and therefore is less efficient at shifting the receptor from its low to high affinity state. This is reflected in the binding curve of xanomeline as more G protein is required to shift the binding curve relative to ACh. Furthermore, the shift from low to high affinity of xanomeline is smaller compared to the full agonist acetylcholine at the M2 mAChR. **C**) At the M4 mAChR, less G protein is required to form the high affinity state of ACh, indicating that within a nanodisc environment, the M4 mAChR is more efficient than the M2 mAChR at coupling to G_{i1} protein. **D)** Owing to its increased efficacy at the M4 mAChR, xanomeline is more efficient at promoting the high affinity state in combination with the G protein at the M4 mAChR. The shift from low to high affinity is greater than that at the M2 mAChR (B) and similar to the full agonist acetylcholine at the M4 mAChR (C). Data represent the mean ± S.E.M. from N=3 (M2/M4 xanomeline), 4 (M2 ACh), 7 (M4 ACh) independent experiments performed in duplicate.

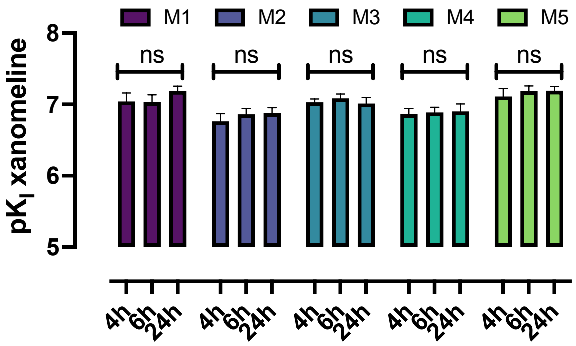

Supplementary Figure 6. Equilibrium binding in CHO cells expressing the M1–M5 mAChR is reached in less than 6h. The plot shows xanomeline affinity (pK_I), as determined by competition binding assays between [^{3}H]-NMS and xanomeline at each of the M1-M5 mAChRs and with three different incubation times: 4h, 6h, and 24h. Data represent the mean ± S.E.M. from a single fit to grouped data from N=3 experiments performed in duplicate. Data were analyzed using a one-site competition model to estimate pK_I of xanomeline. One-way ANOVA multiple comparison analysis; ns (not significant) indicates *P*>0.05.

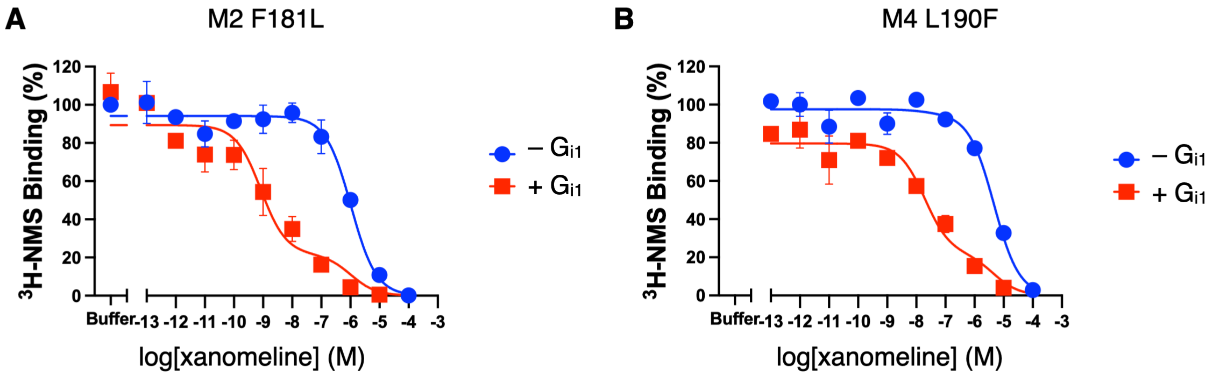

Supplementary Figure 7. Swapping a single ECL2 residue increases xanomeline's binding affinity for the receptor–G protein complex at the M2 mAChR and decreases it at the M4 mAChR. Addition of purified G_{i1}, which favors active-state receptor conformations, increases xanomeline's binding affinity to a greater extent at the M2 F181L mutant mAChR than at the M4 L190F mAChR, as shown by competition for radiolabeled antagonist [^{3}H]-NMS binding to purified, monomeric receptors reconstituted in lipid nanodiscs (+ G_{i1} corresponds to 100 nM G_i). This trend is opposite that observed in the WT receptors (Figure 4), where xanomeline has a substantially higher affinity for the M4 receptor–G protein complex than the M2 receptor–G protein complex. Data represent the mean ± S.E.M. from N=3 independent experiments performed in duplicate. Data were normalized to the buffer-only condition with no G protein.

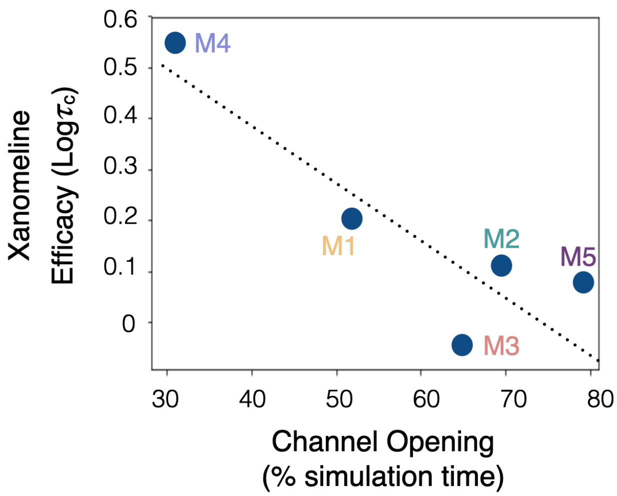

Supplementary Figure 8. Xanomeline signaling efficacy across mAChR subtypes is inversely correlated with channel opening. Channel opening frequency is determined from simulations of the xanomeline-bound active state of each receptor. Efficacy is determined from pERK1/2 assays. The dotted line is a linear fit. The Pearson correlation coefficient is –0.90.

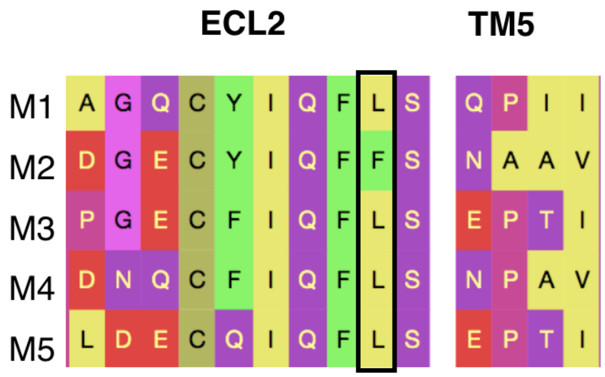

Supplementary Figure 9. Sequence alignment of mAChR subtypes along ECL2 and the top of TM5. The ECL2 position (black box) that we identified as a driver of xanomeline's selectivity between the M2 and M4 mAChRs does not explain xanomeline's behavior at the M3 or M5 mAChRs. The M3 and M5 mAChRs have a leucine at this position like the M4 mAChR. Colored by residue type (Red: anionic, Light Yellow: hydrophobic aliphatic, Green: hydrophobic aromatic, Purple: hydrogen bonding, Pink: Glycine/Proline, Dark Yellow: Cysteine)

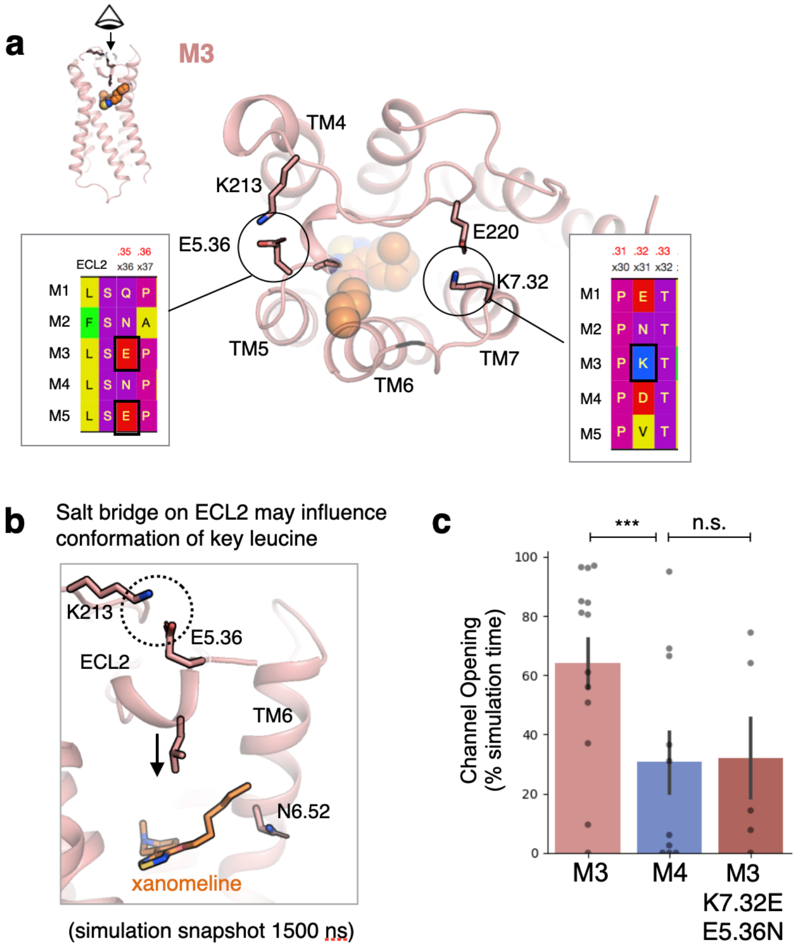

Supplementary Figure 10. Sequence differences in the extracellular loops may explain xanomeline's efficacy-driven selectivity for the M4 mAChR over the M3 and M5 mAChRs. (a) Simulations identify salt bridges formed by extracellular loop 2 of the M3 and M5 mAChR; these salt bridges are not formed in M4. M3 simulation snapshot is pictured with circles highlighting residues in the extracellular vestibule engaged in salt bridges. The salt-bridge between E5.36 and K on ECL2 can form in M5 as well. (b) Simulation snapshot showing how one of these salt bridges at the M3 mAChR, also present at the M5 mAChR, may contribute to a difference in the conformation of the orthosteric binding pocket and xanomeline's binding mode. (c) Mutation of these salt bridges at M3 (K7.32E + E5.36N) ablates the selective effect of xanomeline on channel opening, as observed in simulations of M3 and M4 with bound xanomeline. (M3 WT vs. M4: P=0.014, M3 mutant vs. M4 P>0.05, two-sided Mann-Whitney U-test; N=13 (M3), 10 (M4), 5 (M3 mutant) independent simulations for each WT condition and 5 for mutant condition). Data presented as mean with 68% CI.
