# Supplementary material for: Structural basis of efficacy-driven ligand selectivity at GPCRs
Source: Nat Chem Biol. 2023 Feb 13;19(7):805–14. doi: 10.1038/s41589-022-01247-5 (PMC10299909; doi:10.1038/s41589-022-01247-5)
Supplement: Supplementary file 2 — Reporting Summary [file 41589_2022_1247_MOESM2_ESM.pdf]

nature research

Corresponding author(s): Ron Dror and Celine Valant

Last updated by author(s): Dec 6, 2022

Reporting Summary

Nature Research wishes to improve the reproducibility of the work that we publish. This form provides structure for consistency and transparency in reporting. For further information on Nature Research policies, see our Editorial Policies and the Editorial Policy Checklist.

Statistics

For all statistical analyses, confirm that the following items are present in the figure legend, table legend, main text, or Methods section.

n/a	Confirmed	
☐	☒	The exact sample size (n) for each experimental group/condition, given as a discrete number and unit of measurement
☐	☒	A statement on whether measurements were taken from distinct samples or whether the same sample was measured repeatedly
☐	☒	The statistical test(s) used AND whether they are one- or two-sided<br>*Only common tests should be described solely by name; describe more complex techniques in the Methods section.*
☒	☐	A description of all covariates tested
☐	☒	A description of any assumptions or corrections, such as tests of normality and adjustment for multiple comparisons
☐	☒	A full description of the statistical parameters including central tendency (e.g. means) or other basic estimates (e.g. regression coefficient) AND variation (e.g. standard deviation) or associated estimates of uncertainty (e.g. confidence intervals)
☐	☒	For null hypothesis testing, the test statistic (e.g. F, t, r) with confidence intervals, effect sizes, degrees of freedom and P value noted<br>*Give P values as exact values whenever suitable.*
☒	☐	For Bayesian analysis, information on the choice of priors and Markov chain Monte Carlo settings
☒	☐	For hierarchical and complex designs, identification of the appropriate level for tests and full reporting of outcomes
☐	☒	Estimates of effect sizes (e.g. Cohen's d, Pearson's r), indicating how they were calculated

Our web collection on statistics for biologists contains articles on many of the points above.

Software and code

Policy information about availability of computer code

Data collection	Molecular dynamics (MD) systems were set up using the Schrodinger suite of software tools (Glide, Prime), version 2020-1, ParamChem server (v1), Dabble (2.7.6), Dowser (1999 release), and Gaussian (v16). MD trajectories were performed using the CUDA-enabled version of PMEMD in Amber18 and CPPTRAJ (v16).
Data analysis	MD data analysis was carried out using the Visual Molecular Dynamics (VMD) Python interface(1.9.3), and visualized using the Matplotlib Python package(3.6.2). PyMOL(2.3.2) and VMD(1.9.3) were used for molecular visualization. GraphPad Prism 9.02 was used for other data analysis and curve fitting.

For manuscripts utilizing custom algorithms or software that are central to the research but not yet described in published literature, software must be made available to editors and reviewers. We strongly encourage code deposition in a community repository (e.g. GitHub). See the Nature Research guidelines for submitting code & software for further information.

Data

Policy information about availability of data

All manuscripts must include a data availability statement. This statement should provide the following information, where applicable:
- Accession codes, unique identifiers, or web links for publicly available datasets
- A list of figures that have associated raw data
- A description of any restrictions on data availability

Simulation trajectories are available at https://doi.org/10.5281/zenodo.7407352. Structural models used in this study were accessed from the Protein Data Bank under accession codes 6OIJ (M1, G11), 6OIK (M2, Go), 3UON (M2), and 5DSG (M4). Data supporting the findings of this work are available as the Supplementary Information and Source Data files. Source data are provided with this paper.

Field-specific reporting

Please select the one below that is the best fit for your research. If you are not sure, read the appropriate sections before making your selection.

☒ Life sciences ☐ Behavioural & social sciences ☐ Ecological, evolutionary & environmental sciences

For a reference copy of the document with all sections, see nature.com/documents/nr-reporting-summary-flat.pdf

Life sciences study design

All studies must disclose on these points even when the disclosure is negative.

Sample size	No statistical methods were used to predetermine sample size as sample size was based on availability of resources (including computing time) and on the number of sample repeats required to obtain definitive results.
Data exclusions	No data were excluded from this paper.
Replication	Each experiment was performed 3-6 times. Results were reliably reproducible. At least three simulations were performed under each condition, and simulation results were also reliably reproducible.
Randomization	For simulations, individual conditions were based on the mAChR subtype studied, conformational state of the receptor, and presence or absence of ligands. At least three independent simulations were performed under each condition. The study design could not be randomized as analysis of simulations and experimental results required knowledge of the identity of each sample. Randomization was not relevant to the other experiments in our study as the cellular assays don't have unknown covariates between samples.
Blinding	Investigators were not blinded as experimental and simulation conditions required the investigators to have knowledge of the identity of each sample.

Reporting for specific materials, systems and methods

We require information from authors about some types of materials, experimental systems and methods used in many studies. Here, indicate whether each material, system or method listed is relevant to your study. If you are not sure if a list item applies to your research, read the appropriate section before selecting a response.

Materials & experimental systems

n/a	Involved in the study	
☒	☐	Antibodies
☐	☒	Eukaryotic cell lines
☒	☐	Palaeontology and archaeology
☒	☐	Animals and other organisms
☒	☐	Human research participants
☒	☐	Clinical data
☒	☐	Dual use research of concern

Methods

n/a	Involved in the study	
☒	☐	ChIP-seq
☒	☐	Flow cytometry
☒	☐	MRI-based neuroimaging

Eukaryotic cell lines

Policy information about cell lines

Cell line source(s)	Chinese Hamster Ovary (CHO) FlpIn cells were purchased from Invitrogen (Carlsbad, CA, catalog number R75807); Hi5 insect and Sf9 insect cells from Expression Systems.
Authentication	None of the cell lines used were authenticated
Mycoplasma contamination	The cell lines were not tested for mycloplasma contamination
Commonly misidentified lines (See ICLAC register)	No commonly misidentified lines were used in this study.
